# Personalized antibiotic selection in periodontal treatment improves clinical and microbiological outputs

**DOI:** 10.3389/fcimb.2023.1307380

**Published:** 2023-12-18

**Authors:** Miglė Žiemytė, Andrés Lopez-Roldan, Miguel Carda-Diéguez, Marta Reglero-Santaolaya, Ana Rodriguez, María D. Ferrer, Alex Mira

**Affiliations:** ^1^Genomics & Health Department, Foundation for the Promotion of Health and Biomedical Research of the Valencian Community (FISABIO) Foundation, Valencia, Spain; ^2^Department of Stomatology, Faculty of Medicine and Dentistry, University of Valencia, Valencia, Spain; ^3^School of Health and Welfare, Jönköping University, Jönköping, Sweden

**Keywords:** xCELLigence, biofilms, personalized medicine, periodontitis, oral bacteria, infection, antibiotic treatment, subgingival plaque

## Abstract

**Introduction:**

Periodontitis is a biofilm-mediated disease that is usually treated by non-surgical biofilm elimination with or without antibiotics. Antibiotic treatment in periodontal patients is typically selected empirically or using qPCR or DNA hybridization methods. These approaches are directed towards establishing the levels of different periodontal pathogens in periodontal pockets to infer the antibiotic treatment. However, current methods are costly and do not consider the antibiotic susceptibility of the whole subgingival biofilm.

**Methods:**

In the current manuscript, we have developed a method to culture subgingival samples *ex vivo* in a fast, label-free impedance-based system where biofilm growth is monitored in real-time under exposure to different antibiotics, producing results in 4 hours. To test its efficacy, we performed a double-blind, randomized clinical trial where patients were treated with an antibiotic either selected by the hybridization method (n=32) or by the one with the best effect in the *ex vivo* growth system (n=32).

**Results:**

Antibiotic selection was different in over 80% of the cases. Clinical parameters such as periodontal pocket depth, attachment level, and bleeding upon probing improved in both groups. However, dental plaque was significantly reduced only in the group where antibiotics were selected according to the *ex vivo* growth. In addition, 16S rRNA sequencing showed a larger reduction in periodontal pathogens and a larger increase in health-associated bacteria in the *ex vivo* growth group.

**Discussion:**

The results of clinical and microbiological parameters, together with the reduced cost and low analysis time, support the use of the impedance system for improved individualized antibiotic selection.

## Introduction

Biofilms are defined as bacterial communities composed of single or various bacterial species attached to each other on biotic/abiotic surfaces and encased in a self-secreted extracellular polymeric substance (EPS) (Seneviratne et al., 2008; Lu et al., 2019). For instance, oral biofilms such as dental plaque are composed of several hundred species firmly attached to the teeth above (supragingival dental plaque) or below the gum line (subgingival dental plaque) (Kuboniwa and Lamont, 2010; Checchi and Pascolo, 2018; Lamont et al., 2018). Oral biofilms are strongly associated with the occurrence and the progression of oral diseases such as dental caries, gingivitis, periodontitis and halitosis (Rosier et al., 2018; Johnston et al., 2021). Gingivitis is a reversible inflammatory lesion resulting from interactions between the subgingival dental plaque and the host’s immune‐inflammatory response, which remains contained within the gingiva and does not extend to the periodontal attachment. Contrarily, periodontitis is a chronic multifactorial inflammatory disease associated with dysbiotic subgingival biofilms and characterized by progressive destruction of the tooth-supporting apparatus, which can eventually lead to its loss (Chapple et al., 2015; Nilsson et al., 2018; Papapanou et al., 2018). In addition, there exist many different factors that contribute enormously to the occurrence and development of this disease, including mechanical disruption of gums, tobacco smoking, pathogen infection, deficient oral hygiene and the failure of immune homeostasis (Darveau, 2010; Aljehani, 2014).

Despite the significant advances in the treatment of periodontal diseases, this pathology continues to increase, being the sixth-most common prevalent condition in the world. In fact, lack of adequate treatment and proper personal hygiene have been suggested as the potential causes of the aggravation of the disease (Marcenes et al., 2013). Although subgingival dental plaque elimination by radicular scrapping reduces inflammation, inflammation may return with time, especially in high-risk individuals. For this reason, the combination of mechanical treatment and systemic antibiotics or antiseptics is commonly used, although the specific cases where this is recommended vary among countries (Chapple et al., 2015; Feres et al., 2015; Isola, 2020). For instance, due to concerns about patient’s health and the impact of overuse of antibiotics in the emergence of resistance, their routine use as an adjunct to subgingival debridement in patients with periodontitis is not recommended by the European Federation of Periodontology (Sanz et al., 2020). Many authors recommend the adjunctive use of specific systemic antibiotics for some patient categories (e.g. generalized periodontitis Stage III in young adults) (Teughels et al., 2020). In order to choose an effective antibiotic therapy in each particular case, different PCR and DNA probe hybridization techniques based on the quantification of periodontal bacteria have been developed. Private laboratories usually use Socransky’s complex-based (purple, red, orange, yellow, and green) detection and quantification of periodontal pathogens, including *Aggregatibacter actinomycetemcomitans, Porphyromonas gingivalis, Tannerella forsythia* and *Treptononema denticola* among others (Ko et al., 2021; Van der Weijden et al., 2021). However, these techniques are expensive and only consider the presence of specific bacterial species rather than the antibiotic effect on the whole biofilm (where some bacterial strains can be susceptible and others resistant to a given antibiotic) (Heller et al., 2011). Moreover, these molecular genetic techniques overlook the interaction between different communities and the presence of EPS which can protect biofilm-embedded bacteria (Jiao et al., 2014; Lu et al., 2020; Jakubovics et al., 2021). Thus, all mentioned limitations of currently used methods for antibiotic selection for periodontal disease can lead to treatment failure and consequently to other serious health complications, including bacteremia, pre-mature birth, cardiovascular diseases, or autoimmune diseases, among others (Li et al., 2000; Irshad et al., 2020). Therefore, there is a need to develop new, faster, inexpensive, and more reliable methodologies able to predict the best individualized antibiotic treatment for patients with periodontal disease, in those cases and countries where this is appropriate.

In the current manuscript, we used Real-Time Cell Analysis (RTCA) to evaluate the *in vitro* antibiotic effect on periodontal biofilm growth dynamics of 64 patients with severe periodontal disease. This method consists of growing fresh periodontal samples in a microcosm model where biofilm growth is monitored in real time using impedance-based measurements (Ferrer et al., 2007; Matilla-Cuenca et al., 2020). After that, in order to assess the efficacy of antibiotic use based on this method, a clinical trial was performed where half of the patients were treated with the antibiotic selected by this growth-based system, while the other half were treated with the antibiotic suggested by a standard methodology (hybridization-based antibiotic selection). One and two months after the antibiotic treatment, we compared the disease evolution by evaluation of clinical parameters (including periodontal pocket depth, clinical attachment loss, bleeding on probing and presence of plaque) and changes in subgingival plaque microbiological composition using 16S rRNA gene Illumina sequencing.

## Materials and methods

### Sample collection, transportation, and storage

To select the most suitable transport media and optimal storage time for periodontal biofilm samples, subgingival plaque from 5 individuals was collected by introducing 10 sterile paper-points (size 20) into the deepest subgingival pockets (≥ 6 mm) for 30-60 seconds and placed into 2 mL of three different transport media (**RTF**: Reduced Transport Fluid, **VMG III**: Viability Medium Goteborg without agar or agarose and **VMG III-Agar:** Viability Medium Goteborg with agar and agarose) that were prepared following Dahlen et al. (Dahlén et al., 1993). Samples were then immediately transported from the dental clinic to the laboratory and stored at room temperature for 24 and 48 h to determine the optimal conditions for their storage and recovery, based on bacterial composition similarity between the sample and the biofilm grown (**Supplementary Figure 1**).

### Study design and patients’ selection

To evaluate the use of the RTCA system in antibiotic selection for the individualized treatment of patients with periodontal disease, and to compare the *in vivo* effect of those antibiotics to the current antibiotic selection techniques, a randomized, double blind, parallel group clinical study was designed. The study protocol was reviewed and approved by the Ethics Committee of the University of Valencia (Spain) (H1547805836517). The study was conducted from September 2019 to May 2022.

Required sample size was calculated by a power estimation based on the differences in clinical attachment level (CAL) of 1 mm between the groups with variation in the population of 0.5, a confidence level of 80% and a power of 95%, which suggested a sample size of 20 individuals per group. Due to the length of the study and the multiple visits, a high dropout rate (>15%) was expected. Therefore, a final sample size of over 60 volunteers fulfilling the inclusion/exclusion criteria was estimated.

Inclusion criteria included: adults between 40 and 70 years old, non-smokers or smokers of less than 10 cigarettes a day, with stage III and IV grade A-B periodontitis, presence of at least 20 teeth in the mouth, including at least one molar per quadrant and good general health. Exclusion criteria included: smokers of more than 10 cigarettes a day, patients who have been treated with periodontal therapy in the last 12 months, patients who have been taking antibiotics in the last three months or who routinely used antiseptics in the previous month, patients who due to their systemic condition take antibiotics, pregnant or lactating woman, patients who take medication that might alter the immune system or bone metabolism, patients who are allergic to the antibiotics used in the study and people with diabetes with more than 7% glycosylated hemoglobin. After signing the informed consent form, clinical periodontal indexes such as bleeding on probing (BOP), probing depth (PD), clinical attachment level (CAL) and plaque presence (PLAQUE) were evaluated as previously described (O’Leary et al., 1972; Ainamo and Bay, 1975; Weinberg and Eskow, 2003). Following visits to the dental clinic are indicated in the flow diagram of the study design (**Supplementary Figure 2**).

After the evaluation of clinical parameters, the subgingival plaque samples of 64 volunteers were collected into VMG III transport media, as described above, for both biofilm growth monitoring in real-time (Real-Time culture; RT-Culture) using the xCELLigence impedance monitoring system (Agilent Technologies) and for bacterial composition determination before treatment. In parallel, subgingival plaque samples from the same volunteers were collected into sterile tubes using micro-IDent^®^ plus11 kit (Hain Lifescience GmbH, Germany) and following the instructions provided by a private laboratory (Echevarne Laboratory, Barcelona, Spain) and sent immediately for PCR and specific probe hybridization-based quantification of 11 of the most common periodontal pathogenic species (*Aggregatibacter actinomycetemcomitants*, *Porphyromonas gingivalis*, *Tannerella forsythia*, *Treponema denticola*, *Prevotella intermedia*, *Peptostreptococcus micros*, *Fusobacterium nucleatum/periodonticum*, *Campylobacter rectus*, *Eubacterium nodatum*, *Eikenelia corrodens* and *Capnocytophaga gingivalis/ochrachea/sputigena*) and the corresponding levels of five bacterial complexes based on Sockranscky’s classification (Urbán et al., 2010; Santigli et al., 2016). Patients were then randomly assigned to one of the two treatment groups: half of the patients (32 volunteers) were treated with the antibiotic selected by the *in vitro* culturing system using impedance-based results, while the other half were treated with the antibiotic selected by the hybridization methodology (32 volunteers).

**Figure 1:**
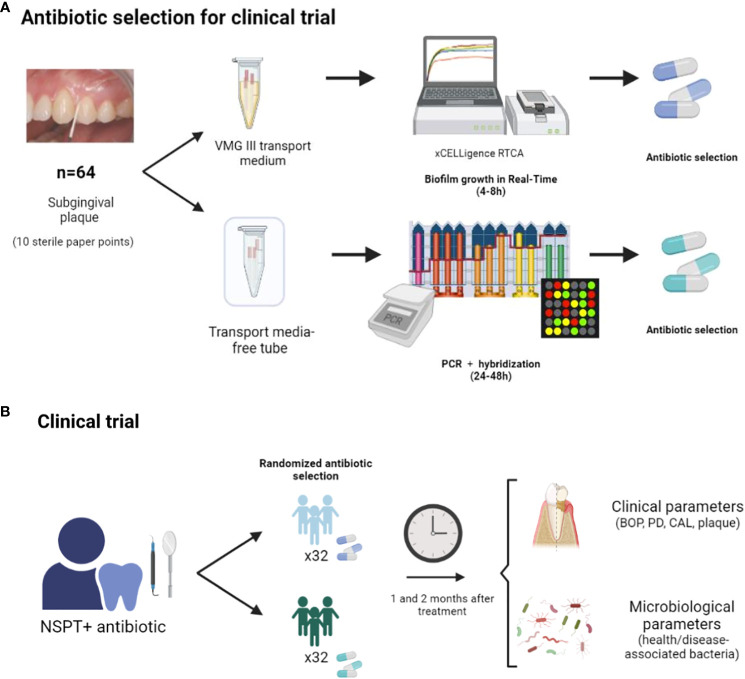
Diagram of the clinical study protocol: subgingival plaque samples from 64 individuals with severe periodontal disease were collected in VMGIII transport media and grown in an impedance system for monitoring periodontal biofilm growth in real time in the presence of antibiotics (Real-Time Culture). Derived biofilms were collected at 8h of growth for DNA extraction and 16S rRNA gene sequencing. In parallel, subgingival plaque samples from these 64 patients were used for standard hybridization-based periodontal pathogen quantification and associated antibiotic recommendations by the German Society of Periodontology (Sanz et al., 2020) **(A)**. For the clinical trial, half of the patients were randomly treated with the antibiotic suggested by the RT-Culture system and the other half with the antibiotic prescribed based on the hybridization of periodontal pathogens. One and two months after antibiotic treatment, the improvement in clinical and microbiological parameters was evaluated for both study groups to assess disease progression **(B)**.

After four and eight weeks of systemic antibiotic treatment, the patient’s oral health was re-evaluated, including all clinical parameters described above (**Supplementary Figure 2**). In addition, new subgingival plaque samples were collected in VMG III transport media, as previously indicated, for the evaluation of the total bacterial composition after antibiotic treatment in both study groups (**Figure 1**). After the treatment, clinical and microbiological parameters were analyzed and compared between methodologies. Three patients of the RT-Culture group were not analyzed, as one of them took other antibiotic prescribed by another professional because of additional infection, and two failed to attend their 2-month reevaluation visit to the dental clinic because of COVID. Similarly, six patients from the hybridization group did not participate in all needed dental clinic visits, and five had incomplete or wrong antibiotic dosing (**Supplementary Figure 3**).

### Randomization and blinding

A randomized, double-blind clinical trial was carried out where patients and dentists were unaware of which methodology was used to select the antibiotic as part of the periodontal therapy. In detail, after consent form approval, dental clinic staff responsible for the prescription of the periodontal antibiotic treatment assigned each patient a code, which was previously generated randomly by another research team member. Depending on the method of treatment selection, this code consisted of a patient number and a letter A or B. Thus, 32 patients were randomly assigned to one method treatment group, while the other 32 were assigned to the other group.

### Real-time biofilm growth monitoring

To select the most suitable transport media for periodontal biofilm samples, the subgingival plaque samples were collected in 2 mL of three different transport media, as indicated above, and homogenized by vortexing for 60 sec. Paper points were discarded, samples were vortexed again for 30 sec and split into three equal aliquots for biofilm growth in real-time either fresh or after 24 and 48 h (t=0, t=24, and t=48) of storage at room temperature. At each time point, the aliquots were centrifuged at 10.000 rpm for 3 minutes, supernatants were discarded, and bacterial pellets were resuspended with 400 µL of BHI supplemented with vitamin K_1_ (VitK_1_) and hemin-menadione (reaching final concentrations of 0.2 mM and 0.5 mM, respectively). Two hundred microliters of the samples were used to monitor biofilm growth in the xCELLigence impedance monitoring system and the other 200 µL of the suspensions were centrifuged, and the pellets stored at -20°C for DNA extraction and subsequent 16S rRNA gene sequencing.

For biofilm growth evaluation of periodontal plaque samples that were stored at room temperature for 0, 24 and 48h (t=0, t=24, and t=48h), the xCELLigence RTCA system was used as previously described (Žiemytė et al., 2020; Žiemytė et al., 2021). Briefly, 100 µL of BHI supplemented with VitK_1_ and hemin-menadione were used for background measurements. Further, 100 µl of the subgingival plaque suspensions of the different time points were added into E-plate wells in duplicate. Then, an overlay of sterile mineral oil was gently added to the top of each well to favor anaerobic conditions. BHI supplemented with VitK_1_ and hemin-menadione without bacterial inoculum was included in each experiment as a negative control. After that, E-plates were incubated in the RTCA system at 37°C, and periodontal biofilm growth was monitored for 8h and 24h, respectively. To analyze the bacterial composition of the formed periodontal biofilms at the bottom of the wells, supernatants were discarded, and biofilms were gently washed with PBS (phosphate buffer saline, pH=7.0) to eliminate unattached bacterial cells. The adhered biofilm was collected using 200 µL of PBS, centrifuged, and the pellet stored at -20°C for further analysis. The same procedure was repeated with the periodontal samples stored at room temperature for 24h and 48h (t=24h and t=48h, respectively) in the different transport media.

### Evaluation of periodontal biofilm susceptibility to antibiotics by real-time monitoring of biofilm growth

To identify the best individual treatment for each patient with severe periodontal disease, three systemic antibiotics commonly used in dental practice, namely amoxicillin, metronidazole and azithromycin (SIGMA), and the combination of amoxicillin and metronidazole, were tested, by evaluating their effect on biofilm formation of the subgingival plaque samples. Similarly to the experiments described above, the subgingival plaque was collected into 2 mL of VMG III transport media and vortexed for 60 seconds. After that, paper-points were discarded, and samples were centrifuged at 10.000 rpm for 3 minutes. Hereafter, the pellet was resuspended with 2 mL of fresh BHI medium supplemented with VitK_1_ and hemin-menadione (Hojo et al., 2007; Mira et al., 2019). All samples were divided into two equal aliquots. A pellet of 1 mL of each sample was used for DNA extraction and 16S rRNA gene sequencing, while the other 1 mL was used for biofilm growth in the RTCA system in the presence of different antibiotics.

To grow periodontal biofilms in the presence of conventional antibiotics in the RTCA system, 100 µL of each antibiotic diluted in BHI medium supplemented with VitK_1_ and hemin-menadione reaching final concentrations of 8 mg/L for amoxicillin, 16 mg/L for metronidazole and 0.4 mg/L for azithromycin, respectively, were used for background measurements. These concentrations correspond to the maximum crevicular gingival fluid concentrations reached after oral administration of each antibiotic (Foulds et al., 1990; Kapoor et al., 2012; Matzneller et al., 2013; Mombelli and Walter, 2019). Then, 100 µL of homogenized periodontal samples were added into the wells, followed by a drop of mineral oil. Then, E-plates were incubated in the RTCA system at 37°C to monitor biofilm growth in real-time. After 8h of growth, supernatants were discarded, and bacterial cells attached to the E-plate surface were gently washed using PBS as described above, and the biofilms collected using 200 µL of PBS. Obtained pellets were stored at -20°C for further analysis. Two replicates of each condition and negative controls were included in each experiment. Biofilm growth dynamics graphs were plotted using the normalized average of the replicates.

### DNA extraction, 16S rRNA gene sequencing and bioinformatic analyses

Genomic DNA from samples of the subgingival dental plaque collected before (initial inocula, t0) and 1 and 2 months after antibiotic treatment (t1 and t2, respectively), and the corresponding biofilms grown in xCELLigence impedance system for eight hours (alone and in combination with antibiotics) was isolated using MagNA Pure LC DNA Isolation Kit III for Bacteria and Fungi (Roche Diagnostics) according to manufacturer’s instructions. The 16S rRNA gene V3-V5 regions were amplified as previously described (Dzidic et al., 2018). The 16S rRNA gene Metagenomic Sequencing Library Preparation Illumina protocol (Part #15044223 Rev. A) was used to prepare an Illumina amplicon library. The amplicons were sequenced using 2x300 bp paired-end sequencing on an Illumina MiSeq Sequencer following the manufacturer’s instructions (Illumina, San Diego, California, USA) at the Sequencing Service in FISABIO Institute (Valencia, Spain).

The obtained reads were analyzed as previously described using dada2 v1.16 (Callahan et al., 2016). Briefly, forward and reverse primers were removed, reads were quality-filtered by end-trimming and by a maximum number of expected errors. After that, reads were dereplicated, paired reads were merged, chimeras and host reads were removed, and the high-quality remaining reads were annotated using SILVA database v138.1 (Quast et al., 2013). The species annotation process in dada2 consists of two steps to ensure validity and robustness in taxonomic assignments. Initially, reads undergo annotation using a Bayesian method, and acceptance is granted only if they achieve a 100% identity match. Subsequently, reads that did not receive species-level annotation in the first step but attained genus-level identification are subjected to a 97% identity filter. The taxonomic assignment is confirmed only if the difference in identity between the best and second-best matches is equal to or greater than 97%.

R programming language was used to compare statistically the proportion of bacteria between groups (CoreTeam, R 2018). Analysis of compositions of microbiomes with bias correction (ANCOM-BC) (Lin and Peddada, 2020) was used to normalize and compare the abundance of bacterial taxa. Multivariate analyses, including principal component analyses (PCA) and canonical correspondence analysis (CCA) were performed using vegan library (Oksanen et al., 2019). PCA loadings were represented using red arrows when indicated and computed as the correlation of the original variables with the first two principal components, whereas coordinates of observations or ‘scores’ were computed as the projection of the original observations on the first two principal components.

Based on the results from previous microbial-based studies, we grouped bacterial species considered periodontal pathogens into “disease-associated”, whereas those that are known to be more abundant in healthy individuals were computed as “health-associated”, following Perez-Chaparro et al. (Pérez-Chaparro et al., 2014). The list of bacteria included in these two groups is enumerated in **Supplementary Table 1**.

### Data availability

Reads have been publicly deposited at the SRA database (accession number: PRJNA892459). All other datasets generated during and/or analysed during the current study are available from the corresponding authors.

## Results

### Evaluation of different transport media and growth conditions for periodontal biofilm cultivation

In order to select the most suitable transport media for biofilm samples from deep periodontal pockets, subgingival biofilms from five volunteers were collected in three different transport media (RTF, VMGIII and VMGIII Agar) and stored at room temperature for 0h, 24h and 48h (**Supplementary Figure 1**). **Supplementary Figure 4A** shows periodontal biofilm growth in real time after the preservation in RTF, VMGIII and VMGIII-Agar transport media as assessed by impedance measurements. The best recovery and biofilm growth were observed in RTF and VMG III media at 0h. However, in contrast to VMGIII, sample storage in RTF media for 24h and 48h resulted in biofilm growth delay and lower total biofilm mass accumulation compared to these parameters at 0h. Similarly, subgingival plaque storage in VMGIII-Agar reduced its capacity to form biofilms, reaching lower CI values compared to those of VMGIII alone in all tested time points. In addition, 16S rRNA gene sequencing was used to compare the initial microbial composition of the inoculum (t=0) to that after 24 and 48h of storage in VMGIII transport media. 16S rRNA gene sequencing data revealed that VMGIII preserved periodontal bacteria diversity for at least 24h (**Supplementary Figure 4B**). Moreover, a similar bacterial composition was observed after 48h of storage, suggesting that VMGIII media could be appropriate for periodontal biofilm transportation. In addition, sequencing data indicated that grown biofilms derived from periodontal samples should be collected at 8h (not 24h) in order to preserve representative periodontal microbiota and avoid the overgrowth of *Streptococcu*s genera (data not shown).

We also performed principal component analysis (PCA) comparing the microbial composition of 24 periodontal samples collected in VMGIII transport media (initial inoculum) to derived biofilms grown in the impedance system collected after 8h (**Supplementary Figure 4C**). Periodontal biofilms grown in the xCELLigence system contained a bacterial composition similar to the initial inocula. Although the increase in *Streptococcus* was observed in some cases, the biofilms grown *in vitro* contained a representative periodontal microbiota, including common periodontal pathogens such as *Tannerella*, *Treponema*, *Filifactor*, *Fusobacterium*, or *Treponema*, among others, as previously described (Mira et al., 2019).

### The effect of conventional antibiotics on periodontal biofilm formation in real-time

**Figure 2:**
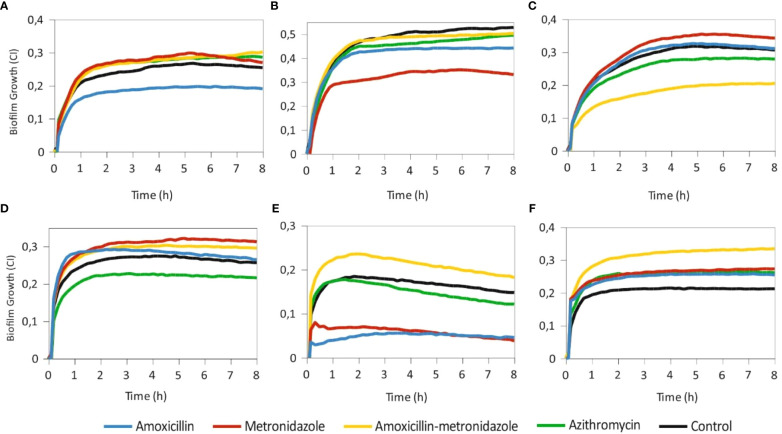
Biofilm growth derived from subgingival plaque samples grown in real time in an impedance-based monitoring system in the presence/absence of amoxicillin, metronidazole, their combination and azithromycin. Antibiotics were added at the beginning of the experiment, along with fresh subgingival plaque samples. Black lines represent antibiotic-free controls. Biofilm growth was registered every 10 minutes for 8 h; SDs are not shown for clarity. Each panel represents a different patient and are representative of cases where one of the antibiotic treatments was effective in reducing biofilm mass compared to the rest (panels **A–D**), where the amoxicillin+metronidazole combination worked worse than each antibiotic alone (panel **E**) or where all antibiotics were ineffective and induced further biofilm growth (panel **F**).

Once the most suitable conditions for periodontal biofilm transportation and growth were established, we grew subgingival biofilms of 64 patients with periodontal disease *in vitro* in the presence of systemic antibiotics commonly used in clinical practice. **Figure 2** illustrates the effect of the tested antibiotics on biofilm formation in six different susceptibility cases. As it can be observed in the figure, periodontal biofilms showed a sample-dependent effect. For example, amoxicillin showed a strong biofilm inhibition capacity in case E and a moderate inhibitory effect in cases A and B, while in other samples, the effect of this antibiotic had no inhibitory effect or even induced biofilm growth compared to the antibiotic-free cell control (case F). Although metronidazole favored biofilm formation in most of the cases (for example, **Figures 2A, C, D, F**), this antibiotic also showed the highest biofilm inhibition capacity in cases B and E. Given that there is compelling evidence that conjunctive antibiotic therapy usually results in better clinical outcomes (Feres et al., 2018), we also evaluated the effect of a combination of both antibiotics (amoxicillin and metronidazole added together) on subgingival biofilm formation. As shown in **Figure 2**, this combination was selected as the most efficient treatment strategy in case C, where periodontal biofilm development was inhibited by almost 50% compared to the untreated control. However, similarly to metronidazole alone, this combination induced biofilm formation in the other cases. Finally, azithromycin, which is effective against many anaerobes including red-complex periodontopathogens, resulted in biofilm inhibition in case D, where other tested antibiotics resulted in biofilm growth induction. However, we have also noted some cases (2/64 of tested cases) where none of the tested antibiotics could halt biofilm formation (**Figure 2F**), suggesting that it might be necessary to test more antibiotics or their combinations in order to select an effective treatment for these patients. Finally, the data show that impedance-based antibiotic selection was consistent between 4 and 8 hours, supporting that suitable antibiotics can normally be chosen in less than four hours (see, for example, **Figure 2E**).

### Antibiotic selection

After determining the most effective antibiotic treatment *in vitro* for each patient with both methodologies (RT-Culture by impedance measurements and hybridization), patients were divided in two groups: 32 patients were given the systemic antibiotic treatment selected by standard hybridization method, and the other 32 patients the antibiotic suggested by the impedance biofilm growth system (**Figure 2**). **Supplementary Figure 5** shows the percentage of cases where each antibiotic was suggested in the two methodologies. The most common antibiotic therapy recommended by the impedance system was azithromycin (41.3% of the cases), while the hybridization method mainly suggested metronidazole (60.4% of cases). In addition, the impedance method recommended amoxicillin in 31.7%, metronidazole in 14.3% and their combination in 12.7% of the cases. In contrast to these results, the hybridization method selected amoxicillin in 15.1% of the cases, amoxicillin and metronidazole combination in 13.2% and azithromycin in 11.3% of the cases. Interestingly, both methodologies suggested the same antibiotic in only in 18.9% of the cases.

### Clinical parameters before and after antibiotic therapy

**Figure 3:**
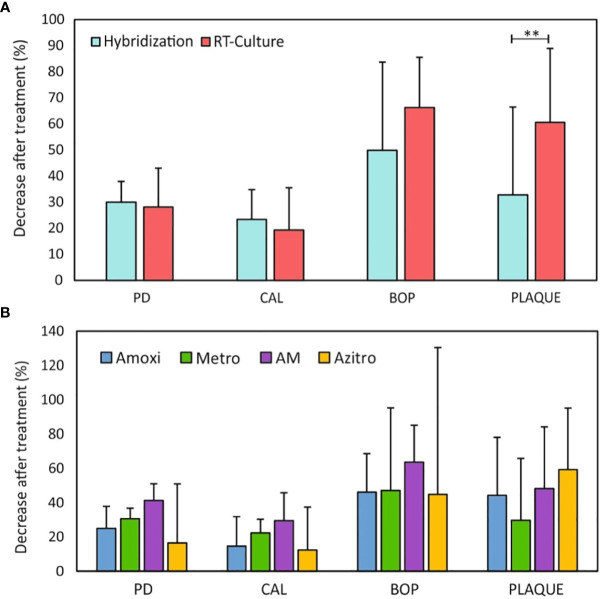
**(A)** Improvement of clinical parameters 2 months after periodontal treatment with antimicrobial therapy suggested by standard hybridization methodology and RT- culture. **(B)** Comparison of clinical parameters improvement 2 months after periodontal treatment among the different systemic antibiotics. Data are represented as a percentage of decrease (improvement) in different clinical parameters. PD - pocket depth; CAL- clinical attachment loss; BOP - bleeding on probing; PLAQUE – plaque presence; Amoxi - amoxicillin; Metro - metronidazole; AM - amoxicillin and metronidazole; Azitro – azithromycin. **p-value < 0.01.

In order to assess the clinical improvement observed in each group after antibiotic treatment, clinical parameters (periodontal pocket depth - PD, clinical attachment level - CAL, bleeding on probing - BOP and plaque presence (PLAQUE)) were evaluated at 2 months after the patients received antibiotic therapy (after treatment, AT) and compared these values to baseline (before treatment, BT). Clinical parameters of patients before and after periodontal treatment in the two groups are shown in **Supplementary Table 2**. **Figure 3A** shows the percentage of improvement of the clinical parameters after treatment with antibiotics selected by standard Hybridization and by culturing the subgingival sample in the impedance system (RT-Culture). All clinical parameters measured improved similarly in both groups, and was highest in BOP, which reached over a 50% reduction in the PCR hybridization group and over 60% reduction in the RT-Culture group. Similarly, plaque presence was reduced considerably in both treatment groups. However, this reduction was significantly higher in the patients who received the antibiotic selected using the impedance-based system in comparison to the hybridization-based methodology. **Supplementary Figure 6A** shows the comparison between evaluated clinical parameters before and after treatment (BT and AT, respectively) for each study group. BOP, PD and CAL were significantly lower after antimicrobial therapy in both-hybridization and RT-Culture groups, while plaque levels were significantly reduced only in the group treated with the antibiotics suggested by the RT-Culture system (*p-value=*0.0001).

**Figure 3B** indicates that all antibiotics reduced all clinical parameters after 2 months of treatment. However, we detected differences in clinical parameters values before and after the treatment depending on the antibiotic used (**Supplementary Figure 6B**). For instance, amoxicillin, metronidazole, and azithromycin resulted in a significant decrease in PD (*p*-value < 0.005), which is a fundamental feature of periodontal disease. Similarly, these antibiotics also resulted in significantly decreased CAL and BOP. On the contrary, only amoxicillin and azithromycin showed a statistically significant decrease in the presence of dental plaque after treatment. Interestingly, no statistically significant decrease was observed in any of the evaluated clinical parameters when the patients were treated with the amoxicillin and metronidazole combination, which is among the most common antimicrobial therapies used to treat periodontitis.

### Changes in microbial composition depending on antibiotic selection methodology and antibiotic treatment

On average, 112,000 16S rRNA reads/sample were sequenced, from which 47,600 reads/sample were annotated at the species level. Using the minimum number of reads annotated to the species level in a sample (22,900 reads), rarefaction analyses were performed, and the curves flattened after 20,000 sequences, showing that bacterial diversity was fully covered.

**Figure 4:**
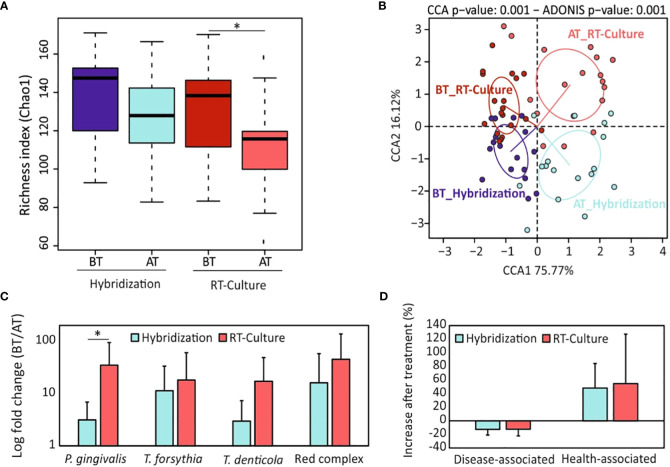
Comparison of microbial composition under different antibiotic selection methodologies. **(A)** Alpha-diversity analysis (bacterial richness Chao1 index) comparison between the two antibiotic selection methods before and two months after treatment. **(B)** Canonical correspondence analyses showing the differences in microbial composition among groups and timepoints. **(C)** Changes in relative abundance of “red complex” periodontal pathogens and their members after treatment (expressed as fold change: before treatment/after treatment) for the two antibiotic selection methods. **(D)** Percentage of increase or decrease in periodontal disease-associated and health-associated bacteria two months after periodontal treatment. BT, before treatment; AT, after treatment; Amoxi, amoxicillin; Metro, metronidazole; AM, amoxicillin and metronidazole; Azitro, azithromycin. *p.value < 0.05.

Chao1 richness index analysis showed that there were no significant differences in the estimated number of bacterial species between both groups before treatment (BT_hybridization vs. BT_RT-Culture) nor after treatment (AT-hybridization vs. AT-RT-Culture). On the other hand, a statistically significant reduction in the number of bacterial species after treatment was observed in the group treated with the antibiotic selected by the impedance system (**Figure 4A**). The CCA plot indicated similarities between the samples before treatment and showed that there were significant differences in the bacterial composition between both groups after antimicrobial therapy (ADONIS *p-value*=0.001) (**Figure 4B**). In addition, the relative abundance of red complex pathogens (*P. gingivalis*, *T. forsythia* and *T. denticola*) after treatment was decreased significantly in both treatment groups (adjusted *p-value*<0.001) (**Supplementary Figure 7A**). Interestingly, when this decrease was compared between both antibiotic selection methodologies, *P. gingivalis* had a significantly higher decrease in the group where the antibiotic treatment was prescribed based on the RT-Culture method compared to hybridization (**Figure 4C**) (*p-value*<0.05). Moreover, 16S rRNA gene sequencing data indicated that periodontal pathogens such as *F. nucleatum*, *Fretibacterium feline*, *Filifactor alocis* and other disease-associated bacteria decreased after the antibiotic treatment independently on the method used (**Figure 4D**; **Supplementary Figure 7B** and **Supplementary Figure 8A**, **Supplementary Tables 2**, **3**). In addition, health-associated bacteria, including *Veillonela, Neisseria*, *Rothia*, *Streptococcus* and other genera increased after treatmet in both groups (adjusted *p-value*<0.001) (**Figure 4D**; **Supplementary Figure 7B**, **Supplementary Figure S8A**, **Supplementary Tables 2**, **3**).

**Figure 5:**
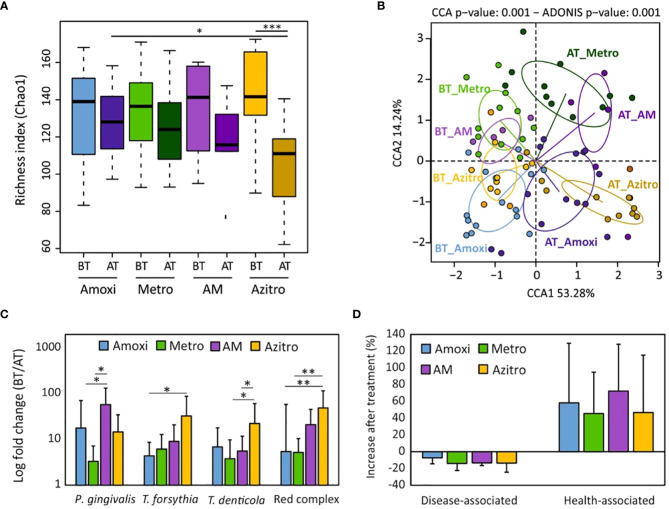
Effect of different systemic antibiotic treatments in microbial composition *in vivo*. **(A)** Alpha-diversity (bacterial richness Chao1 index) analysis for different antibiotic treatment groups. **(B)** Canonical Correspondence Analysis (CCA) of microbial community composition of different treatment groups. **(C)** Changes in abundance of periodontal “red complex” cluster and their members after periodontal treatment and systemic antibiotic use (expressed as fold change: before treatment/after treatment). **(D)** Percentage of increase in disease-associated and health-associated bacteria two months after periodontal treatment for each antibiotic treatment group. BT, before treatment; AT, two months after treatment; Amoxi, amoxicillin; Metro, metronidazole; AM, amoxicillin and metronidazole; Azitro, azithromycin. *p-value<0.05; ** adjusted *p-value*<0.05 and *** adjusted *p-value*<0.001.

Further, we compared whether bacterial richness and composition depended on the antibiotic used. The Chao1 index showed a notable decrease in bacterial richness after treatment with all antibiotics or their combination. However, this decrease was not statistically significant, except in the case of azithromycin (*p-value* < 0.001) (**Figure 5A**). In addition, when we compared the Chao1 index after the treatment among the different antibiotics, a significant decrease in bacterial richness was found in the group treated with azithromycin when compared to amoxicillin. (**Figure 5A**). Bacterial composition was also significantly affected by antibiotic treatment (**Figure 5B**). All tested antibiotics except the amoxicillin metronidazole combination significantly reduced the abundance of red complex members (adjusted *p-value*<0.05) (**Supplementary Figure 9A**). Interestingly, when the reduction of these bacteria was compared between antibiotics, important differences were found. For instance, the combination of amoxicillin and metronidazole decreased *P. gingivalis* levels significantly more than when these two antibiotics were used separately (**Figure 5C**). In addition, *T. forsythia* was significantly more decreased after azithromycin treatment compared to amoxicillin. Similarly, *T. denticola* was significantly more reduced when azithromycin was used in comparison to metronidazole or to the amoxicillin – metronidazole combination (**Figure 5C**). Finally, when these three bacteria were combined into the so-called “red complex”, azithromycin significantly enhanced its reduction in comparison to amoxicillin or metronidazole (adjusted *p-value <*0.005) (**Figure 5C**). On the other hand, both the amoxicillin – metronidazole combination and azithromycin were very efficient not only against red complex pathogens but also against other pathogenic bacteria such as *Fillifactor* or *Alloprevotella* spp (**Supplementary Figure 8B**, **Supplementary Tables 4**, **7**). In contrast to periodontal disease-related pathogens, health-associated bacteria were observed to increase after treatment with all tested antibiotics (**Figure 5D**; **Supplementary Figure 8B**, **Supplementary Figure 9B**).

### Clinical and microbiological outcomes one and two months after antibiotic treatment

**Figure 6:**
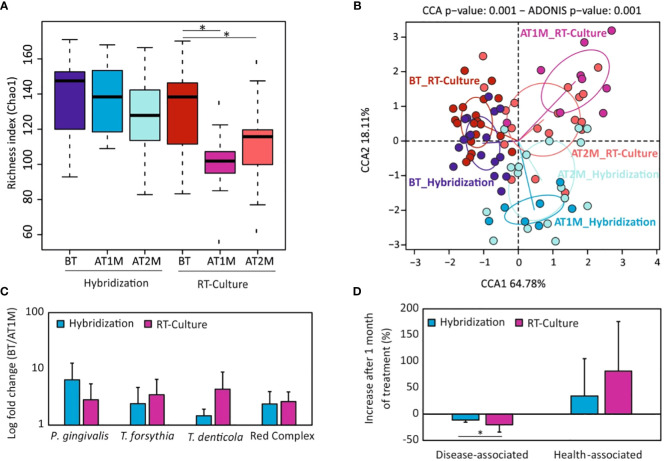
Microbial composition of subgingival plaque samples before and one (AT1) or two months (AT2) after antibiotic treatment. **(A)** Alpha-diversity analysis (bacterial richness Chao1 index) one and two months after antibiotic treatment depending on the antibiotic selection method. **(B)** Canonical correspondence analyses showing the differences in microbial composition among treatment groups and timepoints. **(C)** Changes in abundance of the periodontal “red complex” cluster and their members one month after treatment (expressed as log fold change: before treatment/after treatment). **(D)** Percentage of disease-associated and health-associated bacteria increase or decrease one month after periodontal treatment, for both antibiotic selection methodologies. BT, Before treatment; AT, One month after treatment. *p-value < 0.05.

Clinical evaluation showed that all clinical parameters significantly improved after one month of treatment (BT vs AT1M) in both methodologies (**Supplementary Figure 10**). No significant differences were observed when these parameters were compared between both methodologies. However, PD and CAL were reduced by up to 30% in both groups, while the improvement observed in BOP and plaque parameters was higher in the group treated with antibiotics selected with the RT-Culture system, reaching up to almost 80% reduction (**Supplementary Figure 10**). Further, the results showed that there were no statistically significant differences between the bacterial diversity before and after treatment with antibiotics prescribed by standard hybridization method at both time points (**Figure 6A**). On the contrary, sequencing data in the RT-Culture group showed that bacterial diversity was significantly lower (an indication of improvement in bacterial dysbiosis, as previously outlined by Griffen and collaborators (Griffen et al., 2012)) already at one month of treatment. In addition, even though bacterial diversity slightly increased after two months, the diversity values remained significantly lower compared to the baseline. The CCA plot in **Figure 6B** shows the clustering of samples before and 1 and 2 months after treatment and suggests that microbial composition varied to a greater extent after one month of antibiotic therapy and returned closer to the initial state after 2 months of treatment in both groups. In addition, red complex pathogens also decreased in both treatment groups one month after treatment (**Figure 6C**). Interestingly, a significant reduction in *P. gingivalis* was found only in the RT-Culture group after two months of treatment (*p-value*=0.02) (**Figure 4C**). In general, most periodontal pathogens such as *Fusobacterium*, *Porphyromonas* and others were less abundant after one month of treatment when compared to two months (**Supplementary Figures 11A–C**). However, when the decrease of disease-associated microbes was compared between RT-Culture and hybridization groups after one month of treatment, a significantly reduction was found to be greater in the RT-Culture group (*p-value*=0.02) (**Figure 6D**). In addition, health-associated bacteria increased after one month of treatment in both treatment groups (**Figure 6D** and **Supplementary Figure 12**). Nevertheless, the abundance of health-associated bacteria was significantly higher one month after antibiotic treatment only in the group treated with the RT-Culture methodology (**Supplementary Figure 12**).

## Discussion

Periodontitis is an inflammatory disease that occurs due to prolonged inflammation of the gingiva, accumulation of opportunistic pathogens within subgingival dental plaque, their interaction with the host and the dysbiosis of microbiota embedded in dental biofilms (Zijnge et al., 2010; Jiao et al., 2014; Hajishengallis, 2015). The guidelines for antibiotic use as part of periodontal therapy vary among countries, and previous studies have reported that conventional antibiotics have different effects on periodontitis progression (Heller et al., 2011; Bizzarro et al., 2016; Chapple et al., 2018), emphasizing the need to choose an individualized therapy with the most appropriate antibiotic for each patient, which could result in better clinical outcomes. This has become especially relevant due to the alarming increase in bacterial antibiotic resistance and some organizations strongly discourage antibiotic use in periodontal patients except in specific cases (Sanz et al., 2020). When used, the choice of an antibiotic is normally made empirically, but several molecular techniques exist to select a personalized antibiotic therapy for periodontal patients. These techniques are based on DNA extraction from subgingival samples followed by quantification of periodontal bacteria by either qPCR or DNA hybridization with species-specific probes (Vianna et al., 2005; Horz and Conrads, 2007). Based on the levels of periodontal pathogens, an antibiotic treatment protocol has been proposed (Sanz et al., 2020). However, these techniques have several limitations, such as the heterogeneity in antibiotic susceptibility among strains from the same species (Conrads et al., 2021), and they do not consider the potential effect of the EPS matrix on antibiotic resistance (Heller et al., 2011; Elashiry et al., 2021), or the complex interactions between different bacterial species within biofilms, which could derive in an antibiotic susceptibility pattern which is difficult to predict from bacterial composition (Feres et al., 2015; Villanueva-Castellote et al., 2023). An alternative to molecular methods is the culture of periodontal samples in agar plates in the presence of different antibiotics, in order to establish the overall antibiotic susceptibility of the bacterial community (Rams and Slots, 2023) but there are currently no standardized protocols to culture periodontal samples in biofilms and its efficacy compared to qPCR or molecular probes methodologies needs to be evaluated.

In the current manuscript, we have developed a fast method for culturing fresh periodontal samples with minimal sample processing that allows biofilm growth quantification in real time through impedance measurements. We show that VMGIII transport medium preserved initial bacterial composition and viability to form biofilms for up to 48h. This suggests that periodontal pocket samples could be collected, transferred from the dental clinics and stored in this media at room temperature until further laboratory analysis. In addition, periodontal biofilms grown *in vitro* in the xCELLigence impedance system and collected at 8h showed representative bacterial composition similar to the initial inoculum, suggesting that BHI medium supplemented with VitK_1_ and hemin-menadione is suitable for subgingival biofilm cultivation. It should be noted that under these conditions, *Streptococcus* showed an overgrowth compared to the initial inoculum, possibly due to the sugar concentration of the medium used, suggesting that BHI without sugar, added serum, artificial saliva or another medium (Naginyte et al., 2019) should be used as an alternative for unbiased periodontal biofilm cultivation in future studies.

Impedance-based measurements showed a remarkable variability in antibiotic susceptibility patterns for different patients with periodontal disease, and the antibiotic with best efficacy in reducing biofilm growth could not be predicted from the corresponding bacterial composition. Interestingly, the results in the current manuscript also highlight that amoxicillin and metronidazole combination often resulted in biofilm growth induction (**Figure 6B**) when compared to monotherapy in agreement with recent studies showing that antibiotic combinations often result in reduced antimicrobial efficacy (Lázár et al., 2022). It must be underlined that antibiotic selection by the culturing method agreed with the hybridization method in less than 20% of the cases, indicating that it is very difficult to predict how a whole subgingival biofilm is going to react to a given antibiotic based on the levels of some bacterial species in the sample. In addition, impedance measures were capable of identifying two (2/64) patients for which the periodontal biofilms did not respond to any tested treatment, as all evaluated antibiotics induced biofilm growth, suggesting that other antibiotics or their combinations should be tested.

Half of the patients were prescribed the antibiotic selected by one of the two methods, and we compared the clinical and microbiological outcomes after one and two months in both study groups. The results showed that clinical parameters such as PD, CAL and BOP were improved in both groups after treatment independently of the antibiotic used, and the improvement in BOP was larger in the RT-Culture group (**Figure 3A** and **Supplementary Figure 6A**). However, a significant plaque reduction was only observed in the group treated with antibiotics selected by the impedance system (**Figure 3A** and **Supplementary Figure 6A**). Considering that the amount of plaque is an important factor for the development of the disease, the higher decrease in plaque accumulation in these individuals could represent a significant benefit for periodontal health. Nevertheless, the improvement in plaque accumulation should be confirmed in larger cohorts in the future. Interestingly, the improvement in clinical parameters did not vary between antibiotics (**Figure 3B**), indicating that there was no antibiotic with overall superior performance. This suggests that antibiotic selection should be personalized for optimal effect.

Regarding microbiological parameters, 16S rRNA gene sequencing data showed that bacterial richness was significantly decreased only after the use of antibiotics selected by the RT-Culture system. This is a relevant feature as periodontitis is associated to higher numbers of bacterial species compared to the healthy condition (Camelo-Castillo et al., 2015; Chapple et al., 2018), probably derived from impairment of the immune system and from increased nutrient availability (Mira et al., 2017). The results of our study also indicated that periodontal therapies applied to the tested population decreased periodontal pathogenic taxa such as *Tannerella, Treponema, Fusobacterium*, or *Fillifactor* in both tested groups although the improvement was larger in the RT-Culture methodology (**Figure 4C**; **Supplementary Figure S8A**). *P. gingivalis*, which is considered a keystone periodontal pathogen, was significantly reduced in the group treated with antibiotics suggested by RT-Culture compared with the standard method (**Figure 4C**). In addition, an increase in health-associated taxa, including *Rothia, Neisseria, Veillonella* and others, was observed in both treatment groups, but was only significant at one month in the RT-culture group (**Supplementary Figure 12**, **Supplementary Tables 2**-**8**), Additionally, a significant reduction in disease-associated bacteria was observed only in the RT-Culture group after one month of treatment (**Figure 6D**).

In addition, our results also indicate that antibiotics’ adjunctive influence on the subgingival microbiota depends on the antibiotic (**Figure 5**; **Supplementary Figure 9**). For instance, although all antibiotics inhibited red-complex pathogens the highest inhibition was observed by azithromycin *(p-value<*0.01*)*. Moreover, azithromycin was the only antibiotic which significantly decreased bacterial richness after treatment (**Figure 5A**). Similar findings were also described by other authors who suggested that the use of azithromycin as adjunctive therapy together with radicular scrapping results in improved microbiological outcomes (Haffajee and Socransky, 1994; Oteo et al., 2010; Muniz et al., 2013). Furthermore, the comparison of clinical parameters and microbiological data indicates that most of the patients showed better outcomes one month after antibiotic therapy compared to two months (**Figure 6**; **Supplementary Figures S10**-**12**). This can be related to the ability of periodontal pathogens to persist inside periodontal pockets and regrow, causing inflammation once the treatment is ceased. Similar findings were observed in another study, which investigated the effect of mechanical treatment of subgingival plaque at several timepoints (Johnston et al., 2021). Another study by Bizzaro et al., also found that an antibiotic effect was seen on periodontal microbiota three, but not six months after treatment, suggesting that it is extremely difficult to eradicate periodontal pathogens from deep periodontal pockets (Bizzarro et al., 2016). It is also important to underline that smoking could potentially influence periodontal treatment outcomes, resulting in slower healing or recurring periodontal issues (Leite et al., 2018). However, in this work only 3 and 4 patients were smokers in the hybridization and real-time culture groups, and no significant differences were observed in any of the parameters studied between smokers and non-smokers.

Although many studies have been performed to investigate the critical factors in the occurrence and development of periodontitis and its treatment, to our knowledge, this is the first study which compares personalized antibiotic selection methods evaluating both clinical and microbiological outcomes. Multiple studies have shown that antibiotic use can improve the outcome of periodontal treatment (Feres et al., 2015; Mombelli and Walter, 2019). However, the clinical conditions under which antibiotics should be prescribed in periodontal patients are controversial and vary among countries (Horz and Conrads, 2007; Mombelli and Walter, 2019) and are beyond the scope of the current manuscript. In the cases where antibiotics are used, our data indicate that the use of impedance-based measurements of periodontal biofilms is a reliable antibiotic selection tool. The *in vivo* results indicate that patients treated with an antibiotic selected by this culture method showed equivalent and, in many cases, better clinical and microbiological outcomes when compared to a standard antibiotic selection methodology performed by hybridization technology. In addition, we show that this methodology is more selective, faster (allows the identification of best individual treatment in less than 4h) and cheaper when compared to standard antibiotic selection tools. Thus, we conclude that the Real-Time evaluation of periodontal biofilm growth could improve antibiotic treatment within a personalized dentistry framework. We believe that the use of personalized antibiotic selection in dentistry could not only contribute to a more rationale use of antimicrobials and reduce overall costs of healthcare but might also increase patients’ willingness to treatment and favor the establishment of health-associated microorganisms in subgingival dental plaque (Chapple et al., 2018; Reddy et al., 2019).

## Data availability statement

The data presented in the study are deposited in the SRA repository, accession number PRJNA892459.

## Ethics statement

The studies involving humans were approved by Ethics Committee of the University of Valencia (Spain) (H1547805836517). The studies were conducted in accordance with the local legislation and institutional requirements. The participants provided their written informed consent to participate in this study.

## Author contributions

MŽ: Data curation, Formal analysis, Investigation, Methodology, Writing – original draft. AL-R: Methodology, Supervision, Visualization, Writing – original draft. MC-D: Data curation, Formal analysis, Methodology, Writing – original draft. MR-S: Methodology, Writing – original draft. AR: Methodology, Writing – original draft. MDF: Methodology, Supervision, Visualization, Writing – original draft, Validation, Writing – review & editing. AM: Conceptualization, Funding acquisition, Methodology, Resources, Supervision, Validation, Visualization, Writing – original draft, Writing – review & editing.

## Supplementary material

Supplementary Figure 1 Workflow of experimental set up to evaluate transport media selection and growing conditions. Subgingival plaque samples (n=5) were collected and placed into RTF, VMGIII and VMG III Agar transport media using 10 sterile paper points and stored at room temperature for 0, 24 and 48h. After that, periodontal biofilms were grown in the impedance xCELLigence system, which measures biofilm growth in real-time. Formed biofilms were collected at 8h and 20h for DNA extraction and 16S rRNA gene Illumina sequencing to compare biofilm bacterial composition to the initial subgingival sample inoculum.

Supplementary Figure 2 Flow diagram of the clinical study design. Patients were referred to the University Dental Clinic and evaluated for eligibility criteria. On the first visit, personal data and clinical parameters (probing depth, clinical attachment level, bleeding on probing, and presence of plaque) were recorded, and a complete periapical radiographic series was performed. Subgingival plaque samples were collected with paper points and split in two: One into an empty Eppendorf tube for the quantification of periodontal pathogens by hybridization method; and the other was put in VMG-III transport medium and taken to the lab for evaluation of biofilm growth dynamics in the presence of different antibiotics, followed by determination of the bacterial composition using 16S rRNA gene sequencing. On the second and third visits, non-surgical periodontal treatment (NSPT) was performed, and oral hygiene instructions (OHI) provided. On the third visit, the antibiotic indicated by one of the methodologies was prescribed randomly among patients. At one and two months (fourth and fifth visits), clinical parameters were reevaluated, and subgingival plaque samples were collected for 16S rRNA sequencing to assess the bacterial composition after treatment.

Supplementary Figure 3 Flow diagram of patient selection. Sixty-four patients who met inclusion criteria and agreed to participate in the study were allocated into two groups for antibiotic selection: RT-culture (n=32) and standard, commercially available hybridization (n=32). After the periodontal and antibiotic treatment, clinical and biological parameters were analyzed. Nine patients of the RT-culture group and two patients of the hybridization group were excluded from the statistical analysis when methodologies were compared because both approaches suggested the same antibiotic. In addition, one patient of the RT-culture group was not analysed, as the patient took a different antibiotic prescribed by another professional because of an additional infection and another two failed to attend their 2-month reevaluation visit to the dental clinic because of COVID. Similarly, six patients from the PCR-hybridization group did not attend all needed dental clinic visits, three did not receive antibiotics and five had incomplete antibiotic dosing.

Supplementary Figure 4 **(A)** *In vitro* periodontal biofilm growth in real-time as measured by impedance after preservation of the subgingival plaque in RTF, VMGIII and VMG III-Agar transport media. Samples of periodontal pockets were stored at room temperature for 0h, 24h and 48h. Then, subgingival samples biofilms were grown in the impedance-based monitoring xCELLigence system in real time. Biofilm growth was expressed as Cellular Index (CI), which correlates to total biofilm mass. Data are means of three replicates. SDs are not shown for clarity. **(B)** Bacterial composition in subgingival plaque samples collected from two different periodontal patients after storage in WMGIII transport media for 0, 24 and 48h. **(C)** Principal component analysis of subgingival plaque bacterial composition (“inocula”, blue circles) collected in VMGIII growth media and the derived biofilms grown in the impedance xCELLigence system for 8h (“biofilms”, white circles). The red arrows represent correlations of specific bacteria with the principal components (PC1 and PC2).

Supplementary Figure 5 Percentage of cases where each systemic antibiotic was prescribed by standard hybridization of periodontal pathogens and RT-culture methodologies. Amoxi – amoxicillin, Metro – metronidazole, AM – amoxicillin and metronidazole and Azitro – azithromycin. Black bars indicate cases when both methodologies suggested the use of the same conventional therapy (Shared).

Supplementary Figure 6 **(A)** Comparison of clinical parameters before and after 2 months of treatment. Percentage of examined tooth surfaces (top) and millimeters of pocket depth (bottom) before and after treatment in patients that used the antibiotic determined by hybridization or impedance (RT-culture). and **(B)** comparison in clinical parameters in the same patients, grouped by the antibiotic used. PD - pocket depth; CAL- clinical attachment loss; BOP - bleeding on probing; PLAQUE – plaque presence; BT - before treatment; AT - after treatment; Amoxi-amoxicillin; Metro-metronidazole; AM-amoxicillin and metronidazole; Azitro - azithromycin. * *p-value* < 0.05, ** *p-value* < 0.01 ** *p-value* < 0.005; **ns** - not significant.

Supplementary Figure 7 **(A)** Relative abundance of red complex pathogens (%) before and two months after antibiotic treatment in the groups receiving antibiotics selected by standard hybridization and RT-culture methodologies. **(B)** Relative abundance of disease and health-associated bacteria before and after two months after antibiotic treatment. BT – before treatment; AT - after treatment; * *p.value* < 0.05.

Supplementary Figure 8 Bacterial species mean abundance (%) in each group studied. **(A)** Hybridization vs. RT-Culture system. **(B)** Patients grouped by the antibiotic used, regardless of the antibiotic selection methodology. BT - before treatment; AT – two months after treatment; Amoxi-amoxicillin; Metro-metronidazole; AM-amoxicillin and metronidazole; Azitro-azithromycin.

Supplementary Figure 9 **(A)** Relative abundance (%) of red-complex pathogens two months after periodontal treatment, compared by the prescribed antibiotic group. **(B)** Relative abundance (%) of disease-associated and health-associated bacteria two months after treatment. BT -before treatment; AT - after treatment; Amoxi - amoxicillin; Metro - metronidazole; AM - amoxicillin and metronidazole; Azitro - azithromycin. **p.value* < 0.05.

Supplementary Figure 10 Comparison of clinical parameters improvement (as a percentage of decrease) one and two months after antibiotic treatment according to the antimicrobial therapy (suggested by standard hybridization methodology or RT-culture). PD - pocket depth; CAL- clinical attachment loss; BOP - bleeding on probing; PLAQUE –plaque presence. **p.value* < 0.05.

Supplementary Figure 11 Bacterial species mean abundance (%) in subgingival dental plaque from individuals before treatment **(A)**, after one **(B)** and after two months **(C)** of periodontal treatment in combination with antibiotics. Each column represents a different patient. Only patients for which samples were available at all three timepoints are included.

Supplementary Figure 12 Health-associated species mean abundance (%) in individuals before periodontal treatment (BT) and after 1 (AT1M) or 2 (AT2M) months of treatment, for the two groups of treatment (selected by conventional hybridization and RT-Culture, respectively). **p-value* < 0.05.
