# Supplementary material for: Personalized antibiotic selection in periodontal treatment improves clinical and microbiological outputs
Source: Front Cell Infect Microbiol. 2023 Dec 18;13:1307380. doi: 10.3389/fcimb.2023.1307380 (PMC10765594; doi:10.3389/fcimb.2023.1307380)
Supplement: Supplementary file 1 [file Table_1.docx]

| **Supplementary Table 1.** Health-associated and disease-associated bacterial species in periodontitis. Red-complex-associated periodontal pathogens are marked by asterisks.   \| Health–associated species \| Disease-associated species \| \| --- \| --- \| \| *Actinomyces naeslundii* \| *Fusobacterium nucleatum* \| \| *Actinomyces odontolyticus* \| *Porphyromonas gingivalis* **^⁎^** \| \| *Actinomyces oris* \| *Tannerella forsythia* **^⁎^** \| \| *Corynebacterium durum* \| *Filifactor alocis* \| \| *Corynebacterium matruchotii* \| *Porphyromonas endodontalis* \| \| *Rothia dentocariosa* \| *Parvimonas micra* \| \| *Rothia mucilaginosa* \| *TM7x NA* \| \| *Capnocytophaga sputigena* \| *Selenomonas NA* \| \| *Selenomonas noxia* \| *Campylobacter gracilis* \| \| *Veillonella atypica* \| *Selenomonas sputigena* \| \| *Veillonella dispar* \| *Desulfobulbus NA* \| \| *Veillonella parvula* \| *Treponema denticola* **^⁎^** \| \| *Eikenella corrodens* \| *Fretibacterium fastidiosum* \| \| *Neisseria mucosa* \| *Alloprevotella tannerae* \| \| *Neisseria subflava* \| *[Eubacterium] brachy group brachy* \| \| *Aggregatibacter segnis* \| *Treponema medium* \| \| *Haemophilus parainfluenzae* \| *[Eubacterium] nodatum group NA* \| \|  \| *Selenomonas artemidis* \| \|  \| *Prevotella nigrescens* \| \|  \| *Treponema vincentii* \| \|  \| *Fusobacterium periodonticum* \| \|  \| *Peptostreptococcus stomatis* \| \|  \| *Prevotella intermedia* \| \|  \| *Mogibacterium timidum* \| \|  \| *Aggregatibacter actinomycetemcomitans* \| \|  \| *Anaeroglobus geminatus* \| \|  \| *Campylobacter showae* \| \|  \| *Dialister pneumosintes* \| \|  \| *[Eubacterium] saphenum group saphenum* \| \|  \| *Prevotella denticola* \| \|  \| *Treponema lecithinolyticum* \| |
| --- | --- | --- | --- | --- | --- | --- | --- | --- | --- | --- | --- | --- | --- | --- | --- | --- | --- | --- | --- | --- | --- | --- | --- | --- | --- | --- | --- | --- | --- | --- | --- | --- | --- | --- | --- | --- | --- | --- | --- | --- | --- | --- | --- | --- | --- | --- | --- | --- | --- | --- | --- | --- | --- | --- | --- | --- | --- | --- | --- | --- | --- | --- | --- | --- |
|  |

**Supplementary Table 2.** Clinical parameters at baseline and after two months of non-surgical periodontal treatment in individuals taking an adjunct antibiotic treatment, as recommended by standard hybridization methodology or by real-time culture. Values indicate means and Standard Deviations. P*-values* were assessed using the Wilcoxon test. **PD** - pocket depth; **CAL**- clinical attachment loss; **BOP** - bleeding on probing; **PLAQUE** – plaque presence.

|  | **Hybridization**  **BT** | **Hybridization**  **AT** | **BT/AT**  **adjusted p-value and fold change** | **RT-culture BT** | **RT-culture AT** | **BT/AT**  **adjusted p-value and fold change** |
| --- | --- | --- | --- | --- | --- | --- |
| **PD** | 3.65±0.65 | 2.53±0.38 | 0.0009 (0.69) | 4.09±0.7 | 2.91±0.66 | 0.0002 (0.71) |
| **CAL** | 4.02±0.79 | 3.06±0.64 | 0.0009 (0.76) | 5.26±1.77 | 4.25±1.76 | 0.001 (0.8) |
| **BOP** | 46.35±20.87 | 19.59±10.24 | 0.001  (0.42) | 53.79±21.22 | 17.32±9.33 | 0.0002  (0.32) |
| **PI** | 37.76±34.16 | 25.12±19.9 | 0.15  (0.66) | 63.47±28.42 | 26.47±22.11 | 0.0002  (0.41) |

**Supplementary Table 3**. List of species differentially abundant after 2 months of antibiotic treatment when hybridization method was used as the antibiotic selection treatment.

| **Feature** | **ANCOMBC.pval** | **ANCOMBC.adjpval.fdr** | **BT** | **AT2M** | **log2FC** |
| --- | --- | --- | --- | --- | --- |
| *Treponema NA* | 8.07E-12 | 1.20E-10 | 5.71 | 1.60 | 1.84 |
| *Porphyromonas gingivalis* | 8.72E-09 | 7.48E-08 | 5.44 | 1.76 | 1.63 |
| *Streptococcus NA* | 2.04E-15 | 5.78E-14 | 4.09 | 10.01 | -1.29 |
| *Porphyromonas endodontalis* | 7.93E-08 | 6.07E-07 | 3.84 | 2.63 | 0.55 |
| *Tannerella forsythia* | 8.57E-18 | 3.46E-16 | 3.52 | 0.86 | 2.04 |
| *Filifactor alocis* | 9.78E-14 | 1.98E-12 | 3.50 | 0.81 | 2.10 |
| *Parvimonas micra* | 1.34E-03 | 4.84E-03 | 1.99 | 0.81 | 1.30 |
| *Prevotella NA* | 1.88E-04 | 8.88E-04 | 1.95 | 1.45 | 0.43 |
| *F0058 NA* | 7.84E-14 | 1.71E-12 | 1.94 | 0.76 | 1.36 |
| *Treponema denticola* | 1.79E-09 | 1.75E-08 | 1.86 | 1.06 | 0.81 |
| *Prevotella intermedia* | 1.19E-08 | 9.92E-08 | 1.73 | 0.90 | 0.95 |
| *Desulfobulbus NA* | 9.68E-26 | 1.37E-23 | 1.71 | 0.16 | 3.46 |
| *Leptotrichia NA* | 1.48E-02 | 3.81E-02 | 1.69 | 2.21 | -0.39 |
| *Alloprevotella tannerae* | 4.56E-03 | 1.42E-02 | 1.62 | 2.38 | -0.56 |
| *Fretibacterium NA* | 1.43E-13 | 2.70E-12 | 1.53 | 0.51 | 1.59 |
| *Fretibacterium feline* | 3.72E-33 | 1.05E-30 | 1.21 | 0.13 | 3.25 |
| *Selenomonas sputigena* | 1.16E-03 | 4.37E-03 | 1.20 | 1.04 | 0.21 |
| *Veillonella NA* | 2.32E-09 | 2.19E-08 | 1.10 | 3.37 | -1.62 |
| *Porphyromonas NA* | 7.17E-03 | 2.05E-02 | 1.05 | 0.19 | 2.48 |
| *W5053 NA* | 3.43E-06 | 2.11E-05 | 0.92 | 0.11 | 3.00 |
| *Anaerovoracaceae NA* | 1.81E-25 | 1.71E-23 | 0.87 | 0.14 | 2.63 |
| *[Eubacterium] brachy group brachy* | 3.79E-06 | 2.28E-05 | 0.87 | 0.12 | 2.91 |
| *Fretibacterium fastidiosum* | 3.08E-15 | 7.93E-14 | 0.86 | 0.29 | 1.58 |
| *Prevotella pleuritidis* | 7.25E-09 | 6.41E-08 | 0.86 | 0.22 | 1.98 |
| *Lentimicrobium NA* | 1.03E-06 | 6.76E-06 | 0.69 | 0.66 | 0.07 |
| *Treponema socranskii* | 3.72E-03 | 1.18E-02 | 0.68 | 0.54 | 0.33 |
| *Saccharimonadales NA* | 1.74E-05 | 9.84E-05 | 0.68 | 0.21 | 1.67 |
| *[Eubacterium] saphenum group saphenum* | 1.46E-11 | 1.96E-10 | 0.64 | 0.05 | 3.64 |
| *Prevotella oris* | 2.88E-05 | 1.51E-04 | 0.63 | 2.54 | -2.02 |
| *Campylobacter gracilis* | 2.14E-05 | 1.16E-04 | 0.62 | 1.26 | -1.02 |
| *Treponema maltophilum* | 7.12E-05 | 3.54E-04 | 0.54 | 0.26 | 1.08 |
| *[Eubacterium] nodatum group NA* | 4.96E-10 | 5.20E-09 | 0.54 | 0.16 | 1.73 |
| *Defluviitaleaceae UCG-011 NA* | 8.22E-11 | 9.69E-10 | 0.43 | 0.11 | 2.00 |
| *Rikenellaceae RC9 gut group NA* | 1.22E-07 | 9.12E-07 | 0.39 | 0.41 | -0.07 |
| *Leptotrichia wadei* | 3.58E-03 | 1.15E-02 | 0.38 | 1.12 | -1.55 |
| *Actinomyces NA* | 1.21E-06 | 7.79E-06 | 0.38 | 1.55 | -2.02 |
| *Johnsonella NA* | 8.33E-04 | 3.47E-03 | 0.37 | 0.20 | 0.93 |
| *Prevotella nigrescens* | 2.16E-03 | 7.36E-03 | 0.34 | 1.39 | -2.01 |
| *Capnocytophaga NA* | 1.05E-03 | 4.12E-03 | 0.34 | 0.92 | -1.42 |
| *Oceanivirga NA* | 4.81E-04 | 2.13E-03 | 0.33 | 0.00 | 12.24 |
| *Flexilinea NA* | 5.97E-22 | 3.38E-20 | 0.32 | 0.06 | 2.47 |
| *Leptotrichia buccalis* | 5.94E-10 | 6.01E-09 | 0.29 | 0.08 | 1.77 |
| *Leptotrichia hofstadii* | 3.75E-05 | 1.89E-04 | 0.28 | 0.05 | 2.51 |
| *Mycoplasma NA* | 4.67E-14 | 1.10E-12 | 0.24 | 0.05 | 2.16 |
| *Peptostreptococcus stomatis* | 3.11E-04 | 1.40E-03 | 0.23 | 0.20 | 0.16 |
| *Phocaeicola abscessus* | 3.80E-09 | 3.47E-08 | 0.23 | 0.08 | 1.52 |
| *Moryella NA* | 3.06E-13 | 5.41E-12 | 0.23 | 0.01 | 4.20 |
| *Haemophilus NA* | 2.14E-05 | 1.16E-04 | 0.22 | 1.18 | -2.41 |
| *Capnocytophaga leadbetteri* | 2.39E-02 | 5.59E-02 | 0.22 | 0.69 | -1.66 |
| *Capnocytophaga ochracea* | 2.29E-02 | 5.44E-02 | 0.21 | 0.57 | -1.44 |
| *Prevotella fusca* | 1.19E-02 | 3.16E-02 | 0.21 | 0.03 | 2.67 |
| *Parvimonas NA* | 8.48E-06 | 4.90E-05 | 0.19 | 0.04 | 2.18 |
| *Prevotella dentalis* | 5.46E-16 | 1.93E-14 | 0.19 | 0.04 | 2.34 |
| *Granulicatella NA* | 4.95E-07 | 3.42E-06 | 0.19 | 0.66 | -1.81 |
| *Butyrivibrio NA* | 2.08E-03 | 7.19E-03 | 0.18 | 0.16 | 0.22 |
| *Solobacterium moorei* | 2.47E-02 | 5.68E-02 | 0.18 | 0.20 | -0.15 |
| *Peptoanaerobacter stomatis* | 9.93E-04 | 3.96E-03 | 0.16 | 0.10 | 0.76 |
| *Family XIII UCG-001 NA* | 2.30E-18 | 1.09E-16 | 0.15 | 0.05 | 1.67 |
| *Acholeplasma NA* | 2.09E-04 | 9.69E-04 | 0.13 | 0.01 | 3.95 |
| *Bulleidia extructa* | 3.03E-03 | 9.86E-03 | 0.13 | 0.03 | 2.03 |
| *Catonella NA* | 2.72E-05 | 1.45E-04 | 0.13 | 0.02 | 2.92 |
| *Prevotella melaninogenica* | 7.68E-03 | 2.15E-02 | 0.12 | 0.33 | -1.41 |
| *Prevotella conceptionensis* | 1.79E-02 | 4.37E-02 | 0.11 | 0.20 | -0.83 |
| *Absconditabacteriales (SR1) NA* | 1.06E-04 | 5.16E-04 | 0.11 | 0.04 | 1.44 |
| *Rothia dentocariosa* | 1.94E-07 | 1.41E-06 | 0.11 | 1.10 | -3.30 |
| *Leptotrichia hongkongensis* | 3.92E-11 | 5.05E-10 | 0.09 | 0.90 | -3.31 |
| *Lautropia mirabilis* | 8.55E-23 | 6.05E-21 | 0.08 | 0.70 | -3.10 |
| *Capnocytophaga gingivalis* | 2.33E-08 | 1.83E-07 | 0.08 | 0.57 | -2.83 |
| *Clostridia vadinBB60 group NA* | 2.31E-08 | 1.83E-07 | 0.08 | 0.09 | -0.18 |
| *Prevotella oulorum* | 5.80E-11 | 7.14E-10 | 0.07 | 0.43 | -2.57 |
| *Prevotella loescheii* | 1.21E-03 | 4.49E-03 | 0.07 | 0.43 | -2.60 |
| *Prevotella maculosa* | 1.68E-02 | 4.22E-02 | 0.07 | 0.20 | -1.53 |
| *Eikenella corrodens* | 3.01E-05 | 1.55E-04 | 0.07 | 0.23 | -1.80 |
| *Stomatobaculum longum* | 2.09E-02 | 5.04E-02 | 0.06 | 0.18 | -1.56 |
| *Olsenella NA* | 6.34E-03 | 1.89E-02 | 0.06 | 0.03 | 1.19 |
| *Pseudoramibacter alactolyticus* | 1.84E-10 | 2.08E-09 | 0.06 | 0.01 | 2.63 |
| *Cardiobacterium hominis* | 1.26E-02 | 3.30E-02 | 0.06 | 0.54 | -3.26 |
| *Capnocytophaga sputigena* | 5.23E-06 | 3.08E-05 | 0.06 | 0.59 | -3.41 |
| *Family XI NA* | 6.90E-03 | 2.01E-02 | 0.05 | 0.00 | 9.60 |
| *Erysipelotrichaceae UCG-006 NA* | 2.83E-07 | 2.00E-06 | 0.05 | 0.01 | 1.67 |
| *Bacteroides heparinolyticus* | 9.88E-03 | 2.69E-02 | 0.05 | 0.01 | 1.70 |
| *Shuttleworthia satelles* | 1.71E-02 | 4.26E-02 | 0.04 | 0.03 | 0.47 |
| *Prevotella saccharolytica* | 1.11E-03 | 4.32E-03 | 0.04 | 0.15 | -1.80 |
| *Prevotella salivae* | 1.35E-03 | 4.84E-03 | 0.04 | 0.20 | -2.24 |
| *Mogibacterium timidum* | 1.10E-11 | 1.56E-10 | 0.04 | 0.01 | 2.66 |
| *Treponema parvum* | 7.03E-04 | 2.97E-03 | 0.04 | 0.02 | 0.96 |
| *Gemella sanguinis* | 1.79E-02 | 4.37E-02 | 0.04 | 0.08 | -1.02 |
| *Prevotella enoeca* | 1.63E-02 | 4.13E-02 | 0.04 | 0.02 | 0.71 |
| *Desulfovibrio NA* | 1.04E-02 | 2.81E-02 | 0.04 | 0.01 | 2.56 |
| *Kingella oralis* | 1.53E-15 | 4.82E-14 | 0.04 | 1.15 | -5.00 |
| *Haemophilus parainfluenzae* | 8.74E-04 | 3.59E-03 | 0.03 | 0.25 | -2.92 |
| *Rothia NA* | 3.65E-10 | 3.97E-09 | 0.03 | 0.23 | -2.92 |
| *Bergeyella NA* | 1.86E-12 | 3.10E-11 | 0.03 | 0.18 | -2.60 |
| *Comamonas NA* | 1.39E-02 | 3.62E-02 | 0.03 | 0.00 | 4.40 |
| *Eggerthia catenaformis* | 5.23E-04 | 2.28E-03 | 0.03 | 0.00 | 2.52 |
| *Olsenella uli* | 1.14E-03 | 4.36E-03 | 0.03 | 0.01 | 2.23 |
| *Actinomyces cardiffensis* | 9.48E-03 | 2.60E-02 | 0.03 | 0.00 | 8.67 |
| *Izemoplasmatales NA* | 5.34E-04 | 2.29E-03 | 0.03 | 0.02 | 0.56 |
| *Pyramidobacter piscolens* | 1.73E-03 | 6.05E-03 | 0.02 | 0.00 | 3.90 |
| *Actinomyces naeslundii* | 7.78E-12 | 1.20E-10 | 0.02 | 0.31 | -3.76 |
| *Atopobium parvulum* | 1.62E-02 | 4.12E-02 | 0.02 | 0.19 | -3.17 |
| *Campylobacter showae* | 6.69E-03 | 1.97E-02 | 0.02 | 0.09 | -2.14 |
| *Actinomyces israelii* | 7.15E-03 | 2.05E-02 | 0.02 | 0.00 | 8.01 |
| *Brachymonas NA* | 2.19E-03 | 7.36E-03 | 0.02 | 0.00 | 2.15 |
| *DNF00809 NA* | 1.74E-04 | 8.34E-04 | 0.01 | 0.00 | 7.76 |
| *Abiotrophia defectiva* | 1.61E-03 | 5.69E-03 | 0.01 | 0.07 | -2.36 |
| *Prevotellaceae UCG-004 NA* | 5.26E-03 | 1.60E-02 | 0.01 | 0.00 | 1.45 |
| *Propionivibrio NA* | 2.79E-06 | 1.75E-05 | 0.01 | 0.00 | 1.95 |
| *Rhodospirillales NA* | 7.50E-03 | 2.12E-02 | 0.01 | 0.00 | 4.55 |
| *Actinomyces oricola* | 5.37E-03 | 1.62E-02 | 0.00 | 0.00 | 3.94 |
| *Granulicatella elegans* | 5.03E-03 | 1.55E-02 | 0.00 | 0.03 | -2.81 |
| *Corynebacterium NA* | 2.18E-02 | 5.22E-02 | 0.00 | 0.01 | -0.60 |
| *[Eubacterium] brachy group NA* | 2.65E-03 | 8.82E-03 | 0.00 | 0.00 | 2.87 |
| *Pseudopropionibacterium propionicum* | 9.50E-07 | 6.40E-06 | 0.00 | 0.06 | -4.45 |
| *Porphyromonadaceae NA* | 2.86E-03 | 9.42E-03 | 0.00 | 0.00 | 3.31 |
| *Amnipila NA* | 9.26E-03 | 2.57E-02 | 0.00 | 0.00 | 4.77 |
| *Actinomyces odontolyticus* | 2.40E-04 | 1.09E-03 | 0.00 | 0.02 | -3.84 |
| *[Eubacterium] coprostanoligenes group NA* | 2.36E-02 | 5.56E-02 | 0.00 | 0.00 | 4.41 |
| *Treponema pectinovorum* | 4.45E-03 | 1.40E-02 | 0.00 | 0.01 | -2.12 |
| *Lactobacillales NA* | 2.46E-02 | 5.68E-02 | 0.00 | 0.01 | -3.82 |
| *Actinomyces oris* | 1.31E-03 | 4.81E-03 | 0.00 | 0.04 | -9.30 |
| *Streptococcus gordonii* | 9.53E-04 | 3.85E-03 | 0.00 | 0.01 | -7.63 |
| *Prevotella oris* | 1.20E-02 | 3.16E-02 | 0.00 | 0.00 | -3.66 |

**Supplementary Table 4.** List of bacterial species that were differentially abundant after 2 months of antibiotic treatment when RT-Culture were used as an antibiotic selection method.

| **Feature** | **ANCOMBC.pval** | **ANCOMBC.adjpval.fdr** | **BT** | **AT2M** | **log2FC** |
| --- | --- | --- | --- | --- | --- |
| *Porphyromonas gingivalis* | 6.46E-15 | 1.49E-13 | 12.98 | 3.18 | 2.03 |
| *Fusobacterium nucleatum* | 5.61E-03 | 1.58E-02 | 11.93 | 9.92 | 0.27 |
| *Treponema NA* | 9.51E-17 | 2.93E-15 | 4.47 | 1.18 | 1.92 |
| *Tannerella forsythia* | 3.27E-11 | 4.76E-10 | 4.19 | 1.44 | 1.54 |
| *Filifactor alocis* | 8.09E-13 | 1.32E-11 | 4.09 | 1.12 | 1.87 |
| *Streptococcus NA* | 5.35E-09 | 5.70E-08 | 4.08 | 10.01 | -1.29 |
| *Porphyromonas endodontalis* | 1.45E-05 | 8.05E-05 | 3.33 | 1.99 | 0.74 |
| *Treponema denticola* | 6.36E-10 | 8.80E-09 | 3.14 | 1.20 | 1.39 |
| *Prevotella intermedia* | 3.87E-05 | 1.88E-04 | 2.21 | 1.05 | 1.07 |
| *Prevotella NA* | 5.46E-05 | 2.48E-04 | 1.73 | 0.96 | 0.86 |
| *Dialister invisus* | 4.44E-04 | 1.64E-03 | 1.46 | 0.75 | 0.97 |
| *F0058 NA* | 7.67E-08 | 6.07E-07 | 1.43 | 0.59 | 1.28 |
| *Fretibacterium NA* | 1.21E-22 | 8.95E-21 | 1.42 | 0.19 | 2.90 |
| *Campylobacter NA* | 1.23E-02 | 3.09E-02 | 1.39 | 1.27 | 0.13 |
| *Desulfobulbus NA* | 1.93E-24 | 2.99E-22 | 1.36 | 0.25 | 2.45 |
| *Fretibacterium feline* | 1.57E-20 | 8.69E-19 | 1.22 | 0.37 | 1.73 |
| *Saccharimonadaceae NA* | 7.19E-08 | 5.86E-07 | 1.15 | 0.90 | 0.35 |
| *W5053 NA* | 3.18E-08 | 2.76E-07 | 1.13 | 0.36 | 1.63 |
| *Alloprevotella tannerae* | 6.08E-03 | 1.68E-02 | 0.96 | 1.17 | -0.28 |
| *Neisseria NA* | 3.45E-04 | 1.33E-03 | 0.86 | 2.03 | -1.23 |
| *Fretibacterium fastidiosum* | 3.82E-13 | 7.05E-12 | 0.79 | 0.28 | 1.49 |
| *Lentimicrobium NA* | 7.58E-13 | 1.31E-11 | 0.77 | 0.15 | 2.32 |
| *Defluviitaleaceae UCG-011 NA* | 1.97E-20 | 9.08E-19 | 0.77 | 0.12 | 2.64 |
| *Treponema socranskii* | 6.18E-08 | 5.19E-07 | 0.70 | 0.35 | 0.99 |
| *Oceanivirga NA* | 4.07E-04 | 1.52E-03 | 0.70 | 0.38 | 0.89 |
| *Anaerovoracaceae NA* | 1.08E-13 | 2.30E-12 | 0.69 | 0.16 | 2.06 |
| *Veillonella NA* | 4.28E-09 | 4.75E-08 | 0.67 | 2.89 | -2.10 |
| *Treponema maltophilum* | 9.30E-09 | 9.20E-08 | 0.64 | 0.28 | 1.20 |
| *Porphyromonas NA* | 1.19E-05 | 7.01E-05 | 0.63 | 0.36 | 0.82 |
| *Prevotella pleuritidis* | 1.01E-04 | 4.31E-04 | 0.47 | 0.38 | 0.31 |
| *Rikenellaceae RC9 gut group NA* | 1.29E-22 | 8.95E-21 | 0.44 | 0.09 | 2.32 |
| *Veillonella parvula* | 7.22E-07 | 4.88E-06 | 0.43 | 1.88 | -2.14 |
| *[Eubacterium] nodatum group NA* | 1.79E-07 | 1.34E-06 | 0.42 | 0.16 | 1.38 |
| *Saccharimonadales NA* | 9.27E-10 | 1.22E-08 | 0.42 | 0.15 | 1.49 |
| *Capnocytophaga NA* | 9.62E-05 | 4.16E-04 | 0.36 | 1.29 | -1.84 |
| *Flexilinea NA* | 1.20E-19 | 4.73E-18 | 0.35 | 0.07 | 2.38 |
| *Mycoplasma NA* | 1.51E-15 | 4.19E-14 | 0.33 | 0.07 | 2.34 |
| *[Eubacterium] brachy group brachy* | 9.44E-03 | 2.51E-02 | 0.32 | 0.71 | -1.14 |
| *Actinomyces NA* | 1.19E-04 | 4.91E-04 | 0.30 | 0.80 | -1.43 |
| *[Eubacterium] saphenum group saphenum* | 1.33E-08 | 1.19E-07 | 0.27 | 0.09 | 1.53 |
| *Porphyromonas pasteri* | 1.05E-02 | 2.74E-02 | 0.25 | 0.58 | -1.19 |
| *Johnsonella NA* | 1.29E-03 | 4.39E-03 | 0.24 | 0.18 | 0.44 |
| *Peptococcus NA* | 1.70E-15 | 4.29E-14 | 0.22 | 0.03 | 2.74 |
| *Parvimonas NA* | 4.22E-03 | 1.26E-02 | 0.22 | 0.04 | 2.51 |
| *Anaeroglobus geminatus* | 3.47E-07 | 2.53E-06 | 0.21 | 0.09 | 1.28 |
| *Moryella NA* | 1.71E-17 | 5.93E-16 | 0.21 | 0.05 | 2.00 |
| *Leptotrichia buccalis* | 1.18E-09 | 1.49E-08 | 0.20 | 0.13 | 0.63 |
| *Granulicatella NA* | 5.12E-07 | 3.64E-06 | 0.19 | 0.99 | -2.38 |
| *Prevotella dentalis* | 3.97E-05 | 1.90E-04 | 0.18 | 0.07 | 1.36 |
| *Haemophilus NA* | 9.33E-06 | 5.62E-05 | 0.18 | 0.77 | -2.11 |
| *Peptostreptococcus stomatis* | 4.64E-03 | 1.35E-02 | 0.17 | 0.14 | 0.26 |
| *Catonella NA* | 1.14E-03 | 3.94E-03 | 0.16 | 0.18 | -0.11 |
| *Phocaeicola abscessus* | 3.16E-09 | 3.65E-08 | 0.16 | 0.02 | 2.65 |
| *Prevotella conceptionensis* | 1.39E-04 | 5.67E-04 | 0.15 | 0.02 | 2.59 |
| *Butyrivibrio NA* | 6.79E-07 | 4.70E-06 | 0.14 | 0.01 | 4.06 |
| *Oribacterium NA* | 2.86E-03 | 8.89E-03 | 0.14 | 0.55 | -1.96 |
| *Lachnospiraceae NA* | 4.78E-03 | 1.37E-02 | 0.13 | 0.04 | 1.77 |
| *Family XIII UCG-001 NA* | 2.32E-13 | 4.58E-12 | 0.12 | 0.05 | 1.36 |
| *Lautropia mirabilis* | 2.16E-24 | 2.99E-22 | 0.10 | 0.99 | -3.27 |
| *Alloprevotella rava* | 2.09E-04 | 8.25E-04 | 0.09 | 0.06 | 0.67 |
| *Clostridia UCG-014 NA* | 2.73E-03 | 8.59E-03 | 0.09 | 0.09 | 0.03 |
| *Abiotrophia defectiva* | 3.53E-03 | 1.06E-02 | 0.08 | 0.13 | -0.67 |
| *Candidatus Pacebacteria NA* | 9.98E-03 | 2.63E-02 | 0.08 | 0.00 | 4.16 |
| *Prevotella melaninogenica* | 3.53E-04 | 1.34E-03 | 0.07 | 0.37 | -2.30 |
| *Acholeplasma NA* | 1.06E-02 | 2.74E-02 | 0.07 | 0.05 | 0.48 |
| *Clostridia vadinBB60 group NA* | 8.88E-08 | 6.83E-07 | 0.07 | 0.02 | 1.98 |
| *Campylobacter concisus* | 2.30E-05 | 1.18E-04 | 0.06 | 0.13 | -1.01 |
| *Prevotella baroniae* | 2.03E-04 | 8.15E-04 | 0.06 | 0.05 | 0.31 |
| *Prevotella oulorum* | 1.12E-02 | 2.86E-02 | 0.06 | 0.40 | -2.75 |
| *Kingella oralis* | 2.50E-06 | 1.54E-05 | 0.06 | 0.35 | -2.59 |
| *Megasphaera micronuciformis* | 2.73E-03 | 8.59E-03 | 0.06 | 0.21 | -1.86 |
| *Pseudoramibacter alactolyticus* | 8.05E-05 | 3.60E-04 | 0.06 | 0.02 | 1.61 |
| *Treponema medium* | 1.10E-06 | 7.24E-06 | 0.06 | 0.00 | 9.86 |
| *Prevotella micans* | 5.96E-03 | 1.67E-02 | 0.05 | 0.20 | -1.86 |
| *Treponema parvum* | 2.35E-05 | 1.18E-04 | 0.05 | 0.02 | 1.24 |
| *Izemoplasmatales NA* | 2.33E-05 | 1.18E-04 | 0.05 | 0.01 | 2.14 |
| *Prevotella fusca* | 1.15E-04 | 4.81E-04 | 0.05 | 0.00 | 4.56 |
| *Wolinella succinogenes* | 3.40E-03 | 1.04E-02 | 0.05 | 0.00 | 9.77 |
| *Capnocytophaga sputigena* | 8.27E-05 | 3.64E-04 | 0.05 | 0.19 | -1.89 |
| *Prevotella loescheii* | 7.28E-09 | 7.47E-08 | 0.05 | 0.89 | -4.12 |
| *Prevotella veroralis* | 2.92E-03 | 9.00E-03 | 0.05 | 0.21 | -2.08 |
| *Absconditabacteriales (SR1) NA* | 2.28E-12 | 3.52E-11 | 0.05 | 0.02 | 1.67 |
| *Capnocytophaga gingivalis* | 9.89E-09 | 9.45E-08 | 0.04 | 0.31 | -2.92 |
| *Rothia NA* | 4.95E-05 | 2.32E-04 | 0.04 | 0.27 | -2.91 |
| *Anaeroglobus NA* | 6.72E-03 | 1.84E-02 | 0.03 | 0.01 | 2.07 |
| *Mogibacterium timidum* | 9.27E-04 | 3.29E-03 | 0.03 | 0.01 | 1.18 |
| *Family XI NA* | 2.45E-03 | 7.97E-03 | 0.03 | 0.01 | 2.52 |
| *Bergeyella NA* | 6.09E-04 | 2.19E-03 | 0.03 | 0.09 | -1.65 |
| *Rothia mucilaginosa* | 3.76E-05 | 1.86E-04 | 0.03 | 0.34 | -3.55 |
| *Selenomonas artemidis* | 1.73E-06 | 1.09E-05 | 0.03 | 0.32 | -3.51 |
| *Prevotella pallens* | 1.95E-02 | 4.77E-02 | 0.02 | 0.09 | -2.05 |
| *Erysipelotrichaceae UCG-006 NA* | 9.12E-03 | 2.45E-02 | 0.02 | 0.01 | 1.27 |
| *Atopobium parvulum* | 1.74E-05 | 9.29E-05 | 0.02 | 0.20 | -3.26 |
| *Pyramidobacter piscolens* | 2.23E-09 | 2.68E-08 | 0.02 | 0.00 | 4.81 |
| *Prevotella salivae* | 1.28E-08 | 1.18E-07 | 0.02 | 0.12 | -2.55 |
| *Gracilibacteria JGI 0000069-P22 NA* | 1.31E-03 | 4.43E-03 | 0.02 | 0.00 | 1.85 |
| *Gemella sanguinis* | 1.35E-06 | 8.72E-06 | 0.02 | 0.14 | -3.11 |
| *Eggerthia catenaformis* | 2.02E-02 | 4.88E-02 | 0.02 | 0.01 | 0.84 |
| *Dialister NA* | 4.55E-04 | 1.66E-03 | 0.01 | 0.00 | 4.98 |
| *Gracilibacteria NA* | 1.03E-03 | 3.61E-03 | 0.01 | 0.00 | 7.80 |
| *Streptococcus gordonii* | 2.33E-03 | 7.68E-03 | 0.01 | 0.05 | -2.14 |
| *Bifidobacterium dentium* | 1.67E-02 | 4.17E-02 | 0.01 | 0.01 | 1.05 |
| *DNF00809 NA* | 1.18E-02 | 3.00E-02 | 0.01 | 0.00 | 4.32 |
| *Veillonella tobetsuensis* | 1.30E-05 | 7.32E-05 | 0.01 | 0.17 | -4.11 |
| *Leptotrichia goodfellowii* | 2.66E-03 | 8.55E-03 | 0.01 | 0.29 | -5.41 |
| *[Eubacterium] saphenum group NA* | 7.15E-03 | 1.94E-02 | 0.01 | 0.00 | 0.47 |
| *Veillonella dispar* | 4.63E-03 | 1.35E-02 | 0.00 | 0.10 | -4.51 |
| *Actinomyces gerencseriae* | 2.52E-04 | 9.85E-04 | 0.00 | 0.09 | -4.38 |
| *Oribacterium sinus* | 1.51E-03 | 5.04E-03 | 0.00 | 0.03 | -2.79 |
| *Pseudopropionibacterium propionicum* | 1.66E-05 | 8.99E-05 | 0.00 | 0.02 | -4.85 |
| *Corynebacterium durum* | 4.69E-03 | 1.35E-02 | 0.00 | 0.03 | -6.07 |
| *Haemophilus parainfluenzae* | 1.24E-05 | 7.13E-05 | 0.00 | 0.18 | -11.54 |
| *Actinobacillus pleuropneumoniae* | 1.96E-02 | 4.77E-02 | 0.00 | 0.03 | -8.72 |
| *Prevotella nanceiensis* | 5.17E-05 | 2.39E-04 | 0.00 | 0.02 | -8.60 |
| *Streptococcus parasanguinis* | 1.75E-02 | 4.32E-02 | 0.00 | 0.01 | -6.84 |

**Supplementary Table 5.** List of bacterial species found differentially abundant after 2 months of amoxicillin treatment.

| **Feature** | **ANCOMBC.pval** | **ANCOMBC.adjpval.fdr** | **BT.Amoxi** | **AT2M.Amoxi** | **log2FC** |
| --- | --- | --- | --- | --- | --- |
| *Treponema NA* | 3.64E-24 | 9.64E-22 | 5.20 | 1.74 | 1.58 |
| *Lautropia mirabilis* | 3.32E-23 | 4.40E-21 | 0.05 | 0.61 | -3.74 |
| *Filifactor alocis* | 4.12E-15 | 3.64E-13 | 3.32 | 1.14 | 1.54 |
| *Rikenellaceae RC9 gut group NA* | 6.52E-14 | 4.32E-12 | 0.47 | 0.13 | 1.91 |
| *Tannerella forsythia* | 2.15E-13 | 1.14E-11 | 4.33 | 2.04 | 1.08 |
| *Fretibacterium feline* | 7.13E-12 | 2.84E-10 | 1.31 | 0.51 | 1.36 |
| *Mycoplasma NA* | 7.51E-12 | 2.84E-10 | 0.21 | 0.09 | 1.22 |
| *Moryella NA* | 5.44E-11 | 1.80E-09 | 0.26 | 0.06 | 2.06 |
| *Desulfobulbus NA* | 1.19E-10 | 3.49E-09 | 1.29 | 0.39 | 1.73 |
| *Lentimicrobium NA* | 1.82E-10 | 4.81E-09 | 0.80 | 0.18 | 2.13 |
| *Fretibacterium NA* | 1.20E-09 | 2.90E-08 | 1.44 | 0.37 | 1.95 |
| *Capnocytophaga gingivalis* | 1.36E-09 | 3.00E-08 | 0.06 | 0.35 | -2.65 |
| *Defluviitaleaceae UCG-011 NA* | 1.98E-09 | 4.03E-08 | 0.87 | 0.23 | 1.93 |
| *Kingella oralis* | 2.18E-09 | 4.13E-08 | 0.04 | 0.26 | -2.90 |
| *Flexilinea NA* | 5.06E-09 | 8.94E-08 | 0.45 | 0.11 | 2.04 |
| *Anaerovoracaceae NA* | 5.67E-09 | 9.40E-08 | 0.72 | 0.23 | 1.65 |
| *Peptococcus NA* | 1.15E-08 | 1.80E-07 | 0.19 | 0.06 | 1.70 |
| *Fretibacterium fastidiosum* | 1.41E-08 | 2.08E-07 | 0.90 | 0.33 | 1.44 |
| *Leptotrichia buccalis* | 1.74E-08 | 2.42E-07 | 0.50 | 0.19 | 1.41 |
| *F0058 NA* | 2.22E-08 | 2.94E-07 | 1.94 | 0.97 | 0.99 |
| *Prevotella oulorum* | 3.07E-08 | 3.88E-07 | 0.07 | 0.36 | -2.35 |
| *Prevotella salivae* | 3.75E-08 | 4.52E-07 | 0.03 | 0.28 | -3.04 |
| *Prevotella pleuritidis* | 6.60E-08 | 7.61E-07 | 0.89 | 0.47 | 0.91 |
| *Treponema medium* | 1.20E-07 | 1.32E-06 | 0.06 | 0.00 | 3.52 |
| *Eikenella corrodens* | 1.24E-07 | 1.32E-06 | 0.08 | 0.23 | -1.58 |
| *Selenomonas artemidis* | 1.92E-07 | 1.96E-06 | 0.06 | 0.33 | -2.35 |
| *Streptococcus NA* | 2.52E-07 | 2.47E-06 | 4.75 | 9.18 | -0.95 |
| *Butyrivibrio NA* | 7.11E-07 | 6.73E-06 | 0.07 | 0.01 | 3.36 |
| *Bergeyella NA* | 8.95E-07 | 8.18E-06 | 0.03 | 0.10 | -1.71 |
| *Treponema parvum* | 1.00E-06 | 8.87E-06 | 0.06 | 0.03 | 1.23 |
| *Capnocytophaga sputigena* | 2.53E-06 | 2.16E-05 | 0.05 | 0.30 | -2.64 |
| *Porphyromonas NA* | 3.41E-06 | 2.82E-05 | 1.12 | 0.48 | 1.23 |
| *[Eubacterium] saphenum group saphenum* | 4.99E-06 | 4.00E-05 | 0.26 | 0.13 | 1.01 |
| *Family XIII UCG-001 NA* | 7.07E-06 | 5.51E-05 | 0.12 | 0.08 | 0.66 |
| *Phocaeicola abscessus* | 7.54E-06 | 5.71E-05 | 0.12 | 0.03 | 2.16 |
| *Rothia NA* | 1.19E-05 | 8.76E-05 | 0.02 | 0.25 | -3.35 |
| *Actinomyces odontolyticus* | 1.33E-05 | 9.53E-05 | 0.00 | 0.01 | -7.29 |
| *Atopobium parvulum* | 1.56E-05 | 1.09E-04 | 0.06 | 0.18 | -1.73 |
| *Porphyromonas gingivalis* | 2.34E-05 | 1.54E-04 | 10.11 | 5.10 | 0.99 |
| *Saccharimonadales NA* | 2.39E-05 | 1.54E-04 | 0.30 | 0.16 | 0.97 |
| *Treponema maltophilum* | 2.39E-05 | 1.54E-04 | 0.46 | 0.28 | 0.72 |
| *Haemophilus NA* | 2.48E-05 | 1.56E-04 | 0.15 | 0.71 | -2.28 |
| *Actinomyces NA* | 2.92E-05 | 1.80E-04 | 0.28 | 0.86 | -1.61 |
| *W5053 NA* | 3.58E-05 | 2.16E-04 | 1.45 | 0.47 | 1.61 |
| *Acholeplasma NA* | 4.86E-05 | 2.86E-04 | 0.15 | 0.02 | 2.92 |
| *Oribacterium asaccharolyticum* | 5.13E-05 | 2.96E-04 | 0.00 | 0.04 | -8.70 |
| *Treponema denticola* | 8.47E-05 | 4.78E-04 | 2.34 | 1.44 | 0.70 |
| *Oceanivirga NA* | 9.93E-05 | 5.48E-04 | 0.88 | 0.51 | 0.77 |
| *Bacteroides NA* | 1.05E-04 | 5.70E-04 | 0.01 | 0.00 | 2.82 |
| *Anaeroglobus geminatus* | 1.12E-04 | 5.88E-04 | 0.13 | 0.07 | 0.78 |
| *Actinomyces naeslundii* | 1.13E-04 | 5.88E-04 | 0.01 | 0.12 | -3.85 |
| *Peptoanaerobacter stomatis* | 1.27E-04 | 6.48E-04 | 0.09 | 0.12 | -0.30 |
| *Campylobacter gracilis* | 1.42E-04 | 7.10E-04 | 0.44 | 1.01 | -1.19 |
| *Bacteria NA* | 2.00E-04 | 9.80E-04 | 0.00 | 0.00 | 3.45 |
| *Bacteroidales NA* | 2.57E-04 | 1.24E-03 | 0.03 | 0.02 | 0.14 |
| *Leptotrichia hongkongensis* | 3.33E-04 | 1.58E-03 | 0.05 | 0.29 | -2.49 |
| *Clostridia UCG-014 NA* | 4.78E-04 | 2.22E-03 | 0.11 | 0.10 | 0.11 |
| *Gracilibacteria NA* | 5.00E-04 | 2.28E-03 | 0.02 | 0.00 | 7.70 |
| *Gemella sanguinis* | 5.64E-04 | 2.53E-03 | 0.02 | 0.07 | -1.99 |
| *Prevotella oris* | 6.55E-04 | 2.89E-03 | 0.47 | 2.22 | -2.25 |
| *Eggerthia catenaformis* | 7.57E-04 | 3.29E-03 | 0.01 | 0.00 | 6.96 |
| *Pseudomonas NA* | 9.08E-04 | 3.88E-03 | 0.00 | 0.01 | -7.22 |
| *Rothia mucilaginosa* | 9.54E-04 | 4.01E-03 | 0.06 | 0.15 | -1.40 |
| *Fusobacterium simiae* | 9.90E-04 | 4.10E-03 | 0.11 | 0.05 | 1.06 |
| *Oribacterium NA* | 1.05E-03 | 4.26E-03 | 0.13 | 0.38 | -1.49 |
| *Family XI NA* | 1.21E-03 | 4.87E-03 | 0.20 | 0.00 | 11.12 |
| *Wolinella succinogenes* | 1.57E-03 | 6.13E-03 | 0.01 | 0.00 | 6.95 |
| *Haemophilus parainfluenzae* | 1.57E-03 | 6.13E-03 | 0.01 | 0.06 | -2.62 |
| *Granulicatella NA* | 1.61E-03 | 6.14E-03 | 0.23 | 0.66 | -1.55 |
| *Prevotella fusca* | 1.62E-03 | 6.14E-03 | 0.12 | 0.02 | 2.62 |
| *[Eubacterium] nodatum group NA* | 1.88E-03 | 7.00E-03 | 0.26 | 0.22 | 0.24 |
| *F0332 NA* | 2.05E-03 | 7.55E-03 | 0.03 | 0.01 | 1.93 |
| *DNF00809 NA* | 2.46E-03 | 8.93E-03 | 0.02 | 0.00 | 4.10 |
| *Lactobacillales NA* | 2.50E-03 | 8.94E-03 | 0.00 | 0.01 | -3.88 |
| *Bacteroidia NA* | 2.59E-03 | 9.13E-03 | 0.00 | 0.00 | 2.77 |
| *Absconditabacteriales (SR1) NA* | 2.96E-03 | 1.03E-02 | 0.06 | 0.02 | 1.37 |
| *Stomatobaculum longum* | 3.07E-03 | 1.06E-02 | 0.05 | 0.11 | -1.29 |
| *Johnsonella ignava* | 3.14E-03 | 1.07E-02 | 0.04 | 0.16 | -2.17 |
| *[Eubacterium] brachy group NA* | 3.22E-03 | 1.08E-02 | 0.00 | 0.00 | 4.73 |
| *Prevotella NA* | 3.38E-03 | 1.12E-02 | 0.78 | 0.77 | 0.03 |
| *Treponema socranskii* | 4.62E-03 | 1.51E-02 | 0.72 | 0.50 | 0.51 |
| *Clostridia vadinBB60 group NA* | 5.67E-03 | 1.83E-02 | 0.06 | 0.03 | 0.88 |
| *Candidatus Pacebacteria NA* | 5.89E-03 | 1.88E-02 | 0.16 | 0.00 | 6.23 |
| *Prevotella melaninogenica* | 6.60E-03 | 2.08E-02 | 0.10 | 0.15 | -0.64 |
| *Prevotella loescheii* | 7.26E-03 | 2.26E-02 | 0.09 | 0.44 | -2.26 |
| *Leptotrichia hofstadii* | 7.61E-03 | 2.32E-02 | 0.28 | 0.11 | 1.40 |
| *Rhodospirillales NA* | 7.62E-03 | 2.32E-02 | 0.01 | 0.00 | 7.20 |
| *Dialister NA* | 8.61E-03 | 2.59E-02 | 0.01 | 0.00 | 4.19 |
| *Catonella NA* | 9.24E-03 | 2.74E-02 | 0.15 | 0.20 | -0.39 |
| *Leptotrichia NA* | 9.30E-03 | 2.74E-02 | 1.48 | 2.14 | -0.53 |
| *Prevotella NA* | 1.02E-02 | 2.96E-02 | 1.71 | 1.41 | 0.28 |
| *Actinomyces oricola* | 1.13E-02 | 3.26E-02 | 0.00 | 0.00 | 5.34 |
| *Odoribacter denticanis* | 1.14E-02 | 3.26E-02 | 0.36 | 0.01 | 4.75 |
| *[Eubacterium] saphenum group NA* | 1.48E-02 | 4.17E-02 | 0.01 | 0.01 | 0.63 |
| *Prevotella conceptionensis* | 1.63E-02 | 4.54E-02 | 0.14 | 0.02 | 2.53 |
| *Leptotrichia massiliensis* | 1.70E-02 | 4.68E-02 | 0.01 | 0.14 | -4.31 |
| *Pseudopropionibacterium propionicum* | 1.73E-02 | 4.72E-02 | 0.00 | 0.01 | -2.15 |
| *Bacteroidales F082 NA* | 1.80E-02 | 4.87E-02 | 0.03 | 0.02 | 0.96 |
| *Prevotella micans* | 1.84E-02 | 4.93E-02 | 0.05 | 0.09 | -0.88 |
| *Izemoplasmatales NA* | 1.86E-02 | 4.93E-02 | 0.03 | 0.03 | -0.17 |

**Supplementary Table 6**. List of bacterial species found differentially abundant after 2 months of metronidazole treatment.

| **Feature** | **ANCOMBC.pval** | **ANCOMBC.adjpval.fdr** | **BT.Metro** | **AT2M.Metro** | **log2FC** |
| --- | --- | --- | --- | --- | --- |
| *Desulfobulbus NA* | 1.20E-31 | 3.10E-29 | 2.10 | 0.13 | 3.99 |
| *Fretibacterium feline* | 3.15E-18 | 4.06E-16 | 1.74 | 0.29 | 2.58 |
| *Family XIII UCG-001 NA* | 6.24E-18 | 5.37E-16 | 0.18 | 0.05 | 1.92 |
| *Anaerovoracaceae NA* | 3.91E-16 | 2.52E-14 | 0.89 | 0.15 | 2.60 |
| *Prevotella dentalis* | 1.11E-14 | 5.72E-13 | 0.32 | 0.06 | 2.41 |
| *Prevotella oris* | 3.20E-13 | 1.37E-11 | 0.63 | 2.36 | -1.91 |
| *Mogibacterium timidum* | 3.97E-12 | 1.46E-10 | 0.06 | 0.01 | 2.91 |
| *Eikenella corrodens* | 6.39E-12 | 2.06E-10 | 0.04 | 0.29 | -2.68 |
| *Flexilinea NA* | 3.39E-11 | 9.72E-10 | 0.35 | 0.07 | 2.37 |
| *[Eubacterium] nodatum group NA* | 8.38E-11 | 2.16E-09 | 0.71 | 0.18 | 1.99 |
| *Tannerella forsythia* | 2.55E-10 | 5.97E-09 | 3.61 | 0.94 | 1.95 |
| *Fretibacterium fastidiosum* | 3.24E-10 | 6.96E-09 | 0.86 | 0.30 | 1.54 |
| *Lautropia mirabilis* | 9.26E-10 | 1.84E-08 | 0.10 | 0.86 | -3.08 |
| *Moryella NA* | 3.04E-09 | 5.61E-08 | 0.26 | 0.02 | 3.92 |
| *Paludibacteraceae F0058 NA* | 4.14E-09 | 7.13E-08 | 1.71 | 0.51 | 1.75 |
| *Erysipelotrichaceae UCG-006 NA* | 5.93E-09 | 9.57E-08 | 0.06 | 0.02 | 1.73 |
| *Rothia dentocariosa* | 6.57E-09 | 9.97E-08 | 0.14 | 1.39 | -3.34 |
| *[Eubacterium] saphenum group saphenum* | 1.15E-08 | 1.65E-07 | 0.71 | 0.02 | 4.95 |
| *Leptotrichia buccalis* | 2.07E-08 | 2.81E-07 | 0.19 | 0.02 | 3.21 |
| *Fretibacterium NA* | 4.09E-08 | 5.28E-07 | 1.07 | 0.47 | 1.20 |
| *Filifactor alocis* | 8.87E-08 | 1.09E-06 | 3.66 | 0.99 | 1.89 |
| *Defluviitaleaceae UCG-011 NA* | 1.31E-07 | 1.51E-06 | 0.37 | 0.05 | 2.77 |
| *Leptotrichia hongkongensis* | 1.34E-07 | 1.51E-06 | 0.14 | 1.10 | -2.97 |
| *Bergeyella NA* | 1.70E-07 | 1.83E-06 | 0.03 | 0.20 | -2.74 |
| *Treponema denticola* | 2.68E-07 | 2.77E-06 | 1.86 | 0.89 | 1.06 |
| *Kingella oralis* | 4.24E-07 | 4.21E-06 | 0.05 | 0.85 | -4.08 |
| *Streptococcus NA* | 4.46E-07 | 4.26E-06 | 4.31 | 9.61 | -1.16 |
| *[Eubacterium] brachy group brachy* | 1.21E-06 | 1.12E-05 | 1.08 | 0.15 | 2.83 |
| *Porphyromonas endodontalis* | 1.82E-06 | 1.62E-05 | 4.44 | 3.39 | 0.39 |
| *Pseudoramibacter alactolyticus* | 7.20E-06 | 6.19E-05 | 0.12 | 0.01 | 2.96 |
| *Campylobacter gracilis* | 1.66E-05 | 1.36E-04 | 0.47 | 1.12 | -1.25 |
| *Peptostreptococcus stomatis* | 1.68E-05 | 1.36E-04 | 0.26 | 0.16 | 0.75 |
| *Mycoplasma NA* | 1.81E-05 | 1.42E-04 | 0.23 | 0.04 | 2.40 |
| *Phocaeicola abscessus* | 2.59E-05 | 1.97E-04 | 0.24 | 0.08 | 1.55 |
| *Treponema NA* | 3.20E-05 | 2.36E-04 | 4.85 | 1.39 | 1.81 |
| *Prevotella saccharolytica* | 4.52E-05 | 3.24E-04 | 0.03 | 0.07 | -1.12 |
| *Prevotella intermedia* | 4.66E-05 | 3.25E-04 | 1.24 | 1.05 | 0.24 |
| *Pseudopropionibacterium propionicum* | 5.27E-05 | 3.58E-04 | 0.00 | 0.09 | -9.78 |
| *Catonella NA* | 8.34E-05 | 5.52E-04 | 0.18 | 0.05 | 1.91 |
| *Rothia NA* | 1.18E-04 | 7.59E-04 | 0.02 | 0.25 | -3.34 |
| *Capnocytophaga gingivalis* | 1.30E-04 | 8.19E-04 | 0.05 | 0.54 | -3.57 |
| *Porphyromonas gingivalis* | 1.71E-04 | 1.05E-03 | 5.13 | 2.04 | 1.33 |
| *Parvimonas NA* | 2.35E-04 | 1.41E-03 | 0.21 | 0.04 | 2.32 |
| *DNF00809 NA* | 3.99E-04 | 2.34E-03 | 0.03 | 0.00 | 8.06 |
| *Actinomyces naeslundii* | 4.20E-04 | 2.41E-03 | 0.04 | 0.32 | -3.08 |
| *Prevotella veroralis* | 4.41E-04 | 2.47E-03 | 0.19 | 0.03 | 2.82 |
| *Propionivibrio NA* | 4.80E-04 | 2.63E-03 | 0.01 | 0.00 | 1.84 |
| *Veillonella NA* | 5.02E-04 | 2.70E-03 | 0.80 | 2.42 | -1.59 |
| *Capnocytophaga sputigena* | 5.17E-04 | 2.72E-03 | 0.05 | 0.80 | -3.99 |
| *Prevotella nigrescens* | 6.02E-04 | 3.11E-03 | 0.39 | 1.57 | -1.99 |
| *Prevotella pleuritidis* | 6.19E-04 | 3.13E-03 | 0.58 | 0.14 | 2.01 |
| *Izemoplasmatales NA* | 7.51E-04 | 3.73E-03 | 0.02 | 0.00 | 5.78 |
| *Treponema pectinovorum* | 8.88E-04 | 4.32E-03 | 0.00 | 0.01 | -5.86 |
| *Saccharimonadales NA* | 9.05E-04 | 4.32E-03 | 1.10 | 0.19 | 2.57 |
| *Leptotrichia hofstadii* | 1.40E-03 | 6.59E-03 | 0.10 | 0.01 | 3.53 |
| *W5053 NA* | 1.59E-03 | 7.31E-03 | 0.94 | 0.15 | 2.61 |
| *Comamonas NA* | 1.64E-03 | 7.42E-03 | 0.04 | 0.00 | 8.73 |
| *Treponema maltophilum* | 1.83E-03 | 8.14E-03 | 0.53 | 0.25 | 1.07 |
| *Streptococcus gordonii* | 1.93E-03 | 8.43E-03 | 0.00 | 0.02 | -7.34 |
| *Corynebacterium durum* | 1.96E-03 | 8.43E-03 | 0.00 | 0.04 | -8.83 |
| *Actinomyces oricola* | 2.09E-03 | 8.86E-03 | 0.01 | 0.00 | 3.47 |
| *Centipeda NA* | 2.18E-03 | 9.05E-03 | 0.56 | 1.17 | -1.06 |
| *Olsenella NA* | 2.47E-03 | 1.01E-02 | 0.08 | 0.03 | 1.64 |
| *Aggregatibacter NA* | 2.67E-03 | 1.06E-02 | 0.10 | 0.07 | 0.63 |
| *Clostridia vadinBB60 group NA* | 2.67E-03 | 1.06E-02 | 0.09 | 0.11 | -0.32 |
| *Absconditabacteriales (SR1) NA* | 2.87E-03 | 1.12E-02 | 0.12 | 0.05 | 1.34 |
| *Prevotella oulorum* | 3.40E-03 | 1.31E-02 | 0.07 | 0.35 | -2.34 |
| *Gemella morbillorum* | 3.49E-03 | 1.33E-02 | 0.50 | 0.89 | -0.82 |
| *Parvimonas micra* | 3.58E-03 | 1.33E-02 | 2.52 | 0.69 | 1.86 |
| *Campylobacter showae* | 3.60E-03 | 1.33E-02 | 0.02 | 0.11 | -2.79 |
| *Olsenella uli* | 6.86E-03 | 2.49E-02 | 0.03 | 0.01 | 2.31 |
| *Actinomyces gerencseriae* | 7.00E-03 | 2.51E-02 | 0.00 | 0.01 | -6.93 |
| *Prevotellaceae UCG-004 NA* | 7.27E-03 | 2.57E-02 | 0.01 | 0.00 | 1.54 |
| *Lentimicrobium NA* | 7.80E-03 | 2.72E-02 | 0.55 | 1.02 | -0.90 |
| *Capnocytophaga leadbetteri* | 7.99E-03 | 2.75E-02 | 0.21 | 0.83 | -2.01 |
| *Rikenellaceae RC9 gut group NA* | 8.35E-03 | 2.84E-02 | 0.38 | 0.58 | -0.61 |
| *Actinomyces NA* | 8.59E-03 | 2.88E-02 | 0.48 | 1.39 | -1.55 |
| *Pyramidobacter piscolens* | 8.82E-03 | 2.92E-02 | 0.03 | 0.00 | 3.58 |
| *Actinomyces odontolyticus* | 8.95E-03 | 2.92E-02 | 0.00 | 0.02 | -7.67 |
| *Brachymonas NA* | 9.74E-03 | 3.14E-02 | 0.02 | 0.00 | 1.95 |
| *Prevotella enoeca* | 1.11E-02 | 3.55E-02 | 0.05 | 0.01 | 2.20 |
| *Rothia mucilaginosa* | 1.25E-02 | 3.94E-02 | 0.29 | 0.09 | 1.64 |
| *Capnocytophaga NA* | 1.34E-02 | 4.17E-02 | 0.31 | 0.64 | -1.04 |
| *[Eubacterium] brachy group NA* | 1.40E-02 | 4.29E-02 | 0.01 | 0.00 | 3.29 |
| *Dialister invisus* | 1.48E-02 | 4.49E-02 | 1.52 | 1.10 | 0.47 |
| *Prevotella micans* | 1.67E-02 | 4.96E-02 | 0.04 | 0.04 | 0.20 |
| *Atopobium NA* | 1.68E-02 | 4.96E-02 | 0.46 | 0.23 | 1.02 |
| *Acholeplasma NA* | 1.69E-02 | 4.96E-02 | 0.06 | 0.01 | 2.80 |

**Supplementary Table 7.** List of bacterial species found differentially abundant after 2 amoxicillin and metronidazole treatment.

| **Feature** | **ANCOMBC.pval** | **ANCOMBC.adjpval.fdr** | **BT.AM** | **AT2M.AM** | **log2FC** |
| --- | --- | --- | --- | --- | --- |
| *Olsenella NA* | 2.88E-30 | 7.09E-28 | 0.04 | 0.00 | 4.02 |
| *Bifidobacterium dentium* | 8.30E-20 | 6.81E-18 | 0.00 | 0.00 | 1.78 |
| *Veillonellales-Selenomonadales NA* | 8.30E-20 | 6.81E-18 | 0.00 | 0.00 | 1.78 |
| *Lautropia mirabilis* | 1.11E-18 | 6.85E-17 | 0.04 | 0.28 | -2.73 |
| *Capnocytophaga gingivalis* | 4.02E-15 | 1.98E-13 | 0.02 | 0.20 | -3.18 |
| *Family XIII UCG-001 NA* | 1.41E-13 | 5.79E-12 | 0.17 | 0.02 | 3.30 |
| *Capnocytophaga leadbetteri* | 3.15E-13 | 1.11E-11 | 0.13 | 1.25 | -3.30 |
| *Johnsonella ignava* | 8.97E-12 | 2.76E-10 | 0.04 | 0.00 | 6.64 |
| *Leptotrichia buccalis* | 3.00E-11 | 8.21E-10 | 0.27 | 0.00 | 9.36 |
| *Veillonella NA* | 6.96E-11 | 1.71E-09 | 0.40 | 2.11 | -2.41 |
| *Flexilinea NA* | 1.48E-10 | 3.30E-09 | 0.33 | 0.02 | 4.19 |
| *Saccharimonadales NA* | 7.93E-10 | 1.63E-08 | 0.87 | 0.15 | 2.54 |
| *Filifactor alocis* | 6.98E-09 | 1.27E-07 | 5.10 | 0.87 | 2.55 |
| *Rothia NA* | 7.20E-09 | 1.27E-07 | 0.00 | 0.25 | -9.27 |
| *Atopobium parvulum* | 8.69E-09 | 1.43E-07 | 0.00 | 0.42 | -10.00 |
| *Clostridia vadinBB60 group NA* | 3.38E-08 | 5.20E-07 | 0.06 | 0.01 | 2.10 |
| *Fretibacterium feline* | 3.83E-08 | 5.54E-07 | 0.48 | 0.01 | 6.08 |
| *Mogibacterium timidum* | 6.22E-08 | 8.50E-07 | 0.07 | 0.00 | 4.94 |
| *Kingella oralis* | 8.20E-08 | 1.06E-06 | 0.02 | 0.51 | -4.69 |
| *Porphyromonas NA* | 9.69E-08 | 1.19E-06 | 0.93 | 0.08 | 3.62 |
| *Porphyromonas gingivalis* | 1.53E-07 | 1.78E-06 | 14.76 | 1.31 | 3.49 |
| *Rikenellaceae RC9 gut group NA* | 1.59E-07 | 1.78E-06 | 0.40 | 0.08 | 2.36 |
| *Campylobacter gracilis* | 2.69E-07 | 2.88E-06 | 0.43 | 1.16 | -1.45 |
| *Bergeyella cardium* | 4.63E-07 | 4.75E-06 | 0.02 | 0.01 | 1.43 |
| *Granulicatella NA* | 1.20E-06 | 1.18E-05 | 0.14 | 1.63 | -3.54 |
| *Family XI NA* | 1.46E-06 | 1.38E-05 | 0.05 | 0.02 | 1.26 |
| *Erysipelotrichaceae UCG-006 NA* | 2.07E-06 | 1.89E-05 | 0.03 | 0.01 | 1.02 |
| *Fretibacterium NA* | 4.30E-06 | 3.78E-05 | 1.57 | 0.21 | 2.93 |
| *Capnocytophaga NA* | 8.08E-06 | 6.58E-05 | 0.23 | 1.55 | -2.75 |
| *Prevotella oris* | 8.14E-06 | 6.58E-05 | 0.65 | 2.84 | -2.12 |
| *Pyramidobacter piscolens* | 8.29E-06 | 6.58E-05 | 0.02 | 0.00 | 3.01 |
| *Mycoplasma NA* | 1.42E-05 | 1.09E-04 | 0.38 | 0.04 | 3.19 |
| *Veillonella parvula* | 1.54E-05 | 1.15E-04 | 0.30 | 1.37 | -2.19 |
| *Prevotella NA* | 2.00E-05 | 1.44E-04 | 1.90 | 1.20 | 0.67 |
| *Defluviitaleaceae UCG-011 NA* | 3.24E-05 | 2.28E-04 | 0.29 | 0.04 | 2.80 |
| *Desulfobulbus NA* | 6.46E-05 | 4.37E-04 | 1.02 | 0.13 | 2.99 |
| *Gemella sanguinis* | 6.57E-05 | 4.37E-04 | 0.01 | 0.07 | -3.20 |
| *Abiotrophia defectiva* | 7.37E-05 | 4.66E-04 | 0.04 | 0.17 | -2.19 |
| *Actinomyces NA* | 7.39E-05 | 4.66E-04 | 0.22 | 0.66 | -1.58 |
| *Prevotella loescheii* | 1.05E-04 | 6.46E-04 | 0.05 | 1.28 | -4.75 |
| *Anaerovoracaceae NA* | 1.53E-04 | 9.20E-04 | 0.95 | 0.08 | 3.55 |
| *Aggregatibacter aphrophilus* | 1.60E-04 | 9.35E-04 | 0.11 | 0.00 | 8.03 |
| *Prevotella salivae* | 1.75E-04 | 1.00E-03 | 0.01 | 0.16 | -4.34 |
| *Prevotella dentalis* | 2.16E-04 | 1.21E-03 | 0.09 | 0.05 | 0.70 |
| *Alloprevotella rava* | 2.84E-04 | 1.53E-03 | 0.19 | 0.04 | 2.31 |
| *Shuttleworthia satelles* | 2.86E-04 | 1.53E-03 | 0.02 | 0.00 | 2.08 |
| *Prevotella intermedia* | 3.21E-04 | 1.66E-03 | 2.80 | 0.21 | 3.74 |
| *Prevotellaceae UCG-004 NA* | 3.23E-04 | 1.66E-03 | 0.01 | 0.00 | 3.79 |
| *Bulleidia extructa* | 3.58E-04 | 1.78E-03 | 0.13 | 0.01 | 4.06 |
| *Olsenella uli* | 3.62E-04 | 1.78E-03 | 0.02 | 0.00 | 5.73 |
| *Aggregatibacter actinomycetemcomitans* | 4.01E-04 | 1.94E-03 | 0.01 | 0.00 | 3.85 |
| *Stomatobaculum longum* | 4.54E-04 | 2.15E-03 | 0.07 | 1.00 | -3.76 |
| *Peptococcus NA* | 5.36E-04 | 2.49E-03 | 0.36 | 0.04 | 3.24 |
| *Veillonella tobetsuensis* | 6.30E-04 | 2.82E-03 | 0.05 | 0.10 | -0.98 |
| *Anaeroglobus NA* | 6.31E-04 | 2.82E-03 | 0.06 | 0.00 | 7.17 |
| *Fretibacterium fastidiosum* | 9.26E-04 | 4.07E-03 | 0.55 | 0.34 | 0.70 |
| *Lachnoanaerobaculum NA* | 9.51E-04 | 4.10E-03 | 0.17 | 0.56 | -1.74 |
| *Prevotella pleuritidis* | 1.45E-03 | 6.16E-03 | 0.17 | 0.01 | 4.34 |
| *[Eubacterium] saphenum group saphenum* | 1.50E-03 | 6.26E-03 | 0.17 | 0.02 | 3.30 |
| *Eikenella NA* | 2.11E-03 | 8.67E-03 | 0.08 | 0.17 | -1.04 |
| *Sphaerochaeta NA* | 2.92E-03 | 1.17E-02 | 0.01 | 0.01 | 0.12 |
| *Absconditabacteriales (SR1) NA* | 2.96E-03 | 1.17E-02 | 0.06 | 0.05 | 0.39 |
| *[Eubacterium] nodatum group NA* | 3.14E-03 | 1.23E-02 | 0.85 | 0.15 | 2.49 |
| *Neisseria NA* | 3.83E-03 | 1.47E-02 | 0.73 | 0.94 | -0.36 |
| *Treponema NA* | 4.02E-03 | 1.51E-02 | 3.55 | 1.52 | 1.22 |
| *Tannerella forsythia* | 4.04E-03 | 1.51E-02 | 4.43 | 0.92 | 2.26 |
| *Pseudopropionibacterium propionicum* | 4.23E-03 | 1.55E-02 | 0.00 | 0.01 | -5.23 |
| *Mogibacterium NA* | 4.67E-03 | 1.69E-02 | 0.00 | 0.00 | -3.36 |
| *Porphyromonas endodontalis* | 4.95E-03 | 1.76E-02 | 3.16 | 1.74 | 0.86 |
| *Slackia exigua* | 6.19E-03 | 2.15E-02 | 0.01 | 0.00 | 2.78 |
| *Moryella NA* | 6.21E-03 | 2.15E-02 | 0.07 | 0.02 | 1.90 |
| *Catonella morbi* | 6.72E-03 | 2.30E-02 | 0.36 | 0.28 | 0.36 |
| *Corynebacterium durum* | 7.48E-03 | 2.49E-02 | 0.00 | 0.07 | -7.47 |
| *[Eubacterium] brachy group brachy* | 7.50E-03 | 2.49E-02 | 0.28 | 0.09 | 1.59 |
| *Haemophilus parainfluenzae* | 7.76E-03 | 2.54E-02 | 0.00 | 0.08 | -7.54 |
| *Cardiobacterium valvarum* | 7.89E-03 | 2.55E-02 | 0.04 | 0.04 | 0.06 |
| *Actinomyces naeslundii* | 8.89E-03 | 2.80E-02 | 0.00 | 0.03 | -6.24 |
| *Prevotella saccharolytica* | 8.89E-03 | 2.80E-02 | 0.02 | 0.18 | -3.24 |
| *Leptotrichia hongkongensis* | 9.13E-03 | 2.84E-02 | 0.06 | 0.44 | -2.83 |
| *Eikenella corrodens* | 1.11E-02 | 3.41E-02 | 0.02 | 0.12 | -2.84 |
| *Peptostreptococcus stomatis* | 1.12E-02 | 3.41E-02 | 0.38 | 0.21 | 0.90 |
| *Treponema denticola* | 1.22E-02 | 3.65E-02 | 3.41 | 1.49 | 1.19 |
| *Bacteroides heparinolyticus* | 1.28E-02 | 3.80E-02 | 0.03 | 0.00 | 6.42 |
| *Prevotella enoeca* | 1.37E-02 | 4.02E-02 | 0.02 | 0.00 | 2.02 |
| *Treponema socranskii* | 1.48E-02 | 4.29E-02 | 0.61 | 0.51 | 0.25 |
| *Prevotella NA* | 1.51E-02 | 4.31E-02 | 1.04 | 1.65 | -0.66 |
| *Phocaeicola abscessus* | 1.60E-02 | 4.53E-02 | 0.23 | 0.11 | 1.09 |
| *Campylobacter NA* | 1.70E-02 | 4.75E-02 | 0.98 | 0.93 | 0.08 |

**Supplementary Table 8.** List of bacterial species found differentially abundant after 2 months of azithromycin treatment.

| **Feature** | **ANCOMBC.pval** | **ANCOMBC.adjpval.fdr** | **BT.Azitro** | **AT2M.Azitro** | **log2FC** |
| --- | --- | --- | --- | --- | --- |
| *Porphyromonas gingivalis* | 2.62E-20 | 7.26E-19 | 9.11 | 0.74 | 3.62 |
| *Treponema NA* | 3.39E-13 | 4.68E-12 | 5.05 | 1.00 | 2.33 |
| *Porphyromonas endodontalis* | 1.06E-04 | 4.12E-04 | 4.34 | 2.68 | 0.70 |
| *Streptococcus NA* | 3.98E-15 | 6.61E-14 | 4.21 | 10.55 | -1.32 |
| *Filifactor alocis* | 2.81E-15 | 5.00E-14 | 3.98 | 0.84 | 2.24 |
| *Tannerella forsythia* | 7.54E-26 | 3.13E-24 | 3.41 | 0.23 | 3.91 |
| *Treponema denticola* | 6.00E-13 | 7.86E-12 | 2.89 | 0.64 | 2.17 |
| *Prevotella intermedia* | 9.85E-06 | 4.89E-05 | 2.47 | 1.26 | 0.97 |
| *Paludibacteraceae F0058 NA* | 5.37E-06 | 2.79E-05 | 2.00 | 0.65 | 1.62 |
| *Fretibacterium NA* | 3.07E-17 | 6.38E-16 | 1.46 | 0.13 | 3.45 |
| *Saccharimonadaceae NA* | 1.66E-11 | 2.06E-10 | 1.43 | 0.25 | 2.52 |
| *Campylobacter NA* | 1.75E-03 | 4.83E-03 | 1.41 | 0.54 | 1.40 |
| *Fretibacterium feline* | 2.42E-37 | 1.51E-35 | 1.37 | 0.04 | 5.07 |
| *Desulfobulbus NA* | 3.18E-181 | 7.91E-179 | 1.31 | 0.00 | 10.35 |
| *Lentimicrobium NA* | 5.89E-54 | 7.33E-52 | 1.23 | 0.02 | 6.09 |
| *Dialister invisus* | 6.12E-03 | 1.51E-02 | 1.13 | 0.60 | 0.91 |
| *Veillonella NA* | 2.41E-08 | 2.00E-07 | 1.02 | 4.78 | -2.23 |
| *Porphyromonas NA* | 5.40E-03 | 1.36E-02 | 0.96 | 0.44 | 1.14 |
| *Fretibacterium fastidiosum* | 5.89E-19 | 1.33E-17 | 0.87 | 0.11 | 2.96 |
| *Selenomonas sputigena* | 1.39E-02 | 2.95E-02 | 0.86 | 0.38 | 1.17 |
| *Neisseria NA* | 2.50E-04 | 8.40E-04 | 0.78 | 2.55 | -1.71 |
| *Family XI W5053 NA* | 2.72E-08 | 2.12E-07 | 0.76 | 0.01 | 6.15 |
| *Defluviitaleaceae UCG-011 NA* | 1.11E-24 | 3.95E-23 | 0.71 | 0.04 | 4.08 |
| *Treponema maltophilum* | 1.21E-05 | 5.78E-05 | 0.67 | 0.23 | 1.55 |
| *Prevotella pleuritidis* | 2.38E-02 | 4.73E-02 | 0.66 | 0.31 | 1.11 |
| *Centipeda NA* | 2.18E-02 | 4.46E-02 | 0.65 | 0.77 | -0.24 |
| *Veillonella parvula* | 3.02E-14 | 4.42E-13 | 0.65 | 2.85 | -2.12 |
| *Anaerovoracaceae NA* | 2.23E-20 | 6.94E-19 | 0.59 | 0.06 | 3.26 |
| *Treponema socranskii* | 3.68E-11 | 4.16E-10 | 0.59 | 0.18 | 1.73 |
| *Oceanivirga NA* | 3.19E-03 | 8.37E-03 | 0.50 | 0.00 | 12.52 |
| *Saccharimonadales NA* | 3.33E-07 | 2.30E-06 | 0.49 | 0.21 | 1.21 |
| *Capnocytophaga NA* | 3.15E-11 | 3.74E-10 | 0.46 | 1.80 | -1.98 |
| *Rikenellaceae RC9 gut group NA* | 9.99E-20 | 2.49E-18 | 0.43 | 0.06 | 2.86 |
| *Mycoplasma NA* | 7.72E-17 | 1.48E-15 | 0.38 | 0.11 | 1.82 |
| *[Eubacterium] saphenum group saphenum* | 1.87E-10 | 2.03E-09 | 0.37 | 0.04 | 3.12 |
| *[Eubacterium] nodatum group NA* | 1.46E-09 | 1.40E-08 | 0.36 | 0.07 | 2.39 |
| *Prevotella nigrescens* | 1.46E-02 | 3.08E-02 | 0.32 | 0.83 | -1.38 |
| *Aggregatibacter NA* | 1.11E-02 | 2.45E-02 | 0.29 | 0.15 | 0.91 |
| *Tannerella NA* | 1.26E-04 | 4.55E-04 | 0.28 | 0.81 | -1.51 |
| *Actinomyces NA* | 5.10E-07 | 3.34E-06 | 0.28 | 1.05 | -1.90 |
| *Johnsonella NA* | 6.28E-03 | 1.51E-02 | 0.24 | 0.21 | 0.23 |
| *Granulicatella NA* | 8.74E-06 | 4.44E-05 | 0.24 | 0.98 | -2.04 |
| *Leptotrichia buccalis* | 6.68E-05 | 2.68E-04 | 0.22 | 0.09 | 1.28 |
| *Flexilinea NA* | 5.32E-45 | 4.42E-43 | 0.21 | 0.01 | 4.36 |
| *Aggregatibacter aphrophilus* | 1.64E-02 | 3.41E-02 | 0.18 | 0.29 | -0.63 |
| *Haemophilus NA* | 2.55E-08 | 2.05E-07 | 0.18 | 1.69 | -3.26 |
| *Peptostreptococcus stomatis* | 6.09E-04 | 1.85E-03 | 0.17 | 0.09 | 0.92 |
| *Leptotrichia hofstadii* | 8.53E-03 | 1.95E-02 | 0.17 | 0.07 | 1.20 |
| *Moryella NA* | 1.81E-33 | 9.02E-32 | 0.16 | 0.01 | 4.73 |
| *Catonella NA* | 9.89E-03 | 2.22E-02 | 0.16 | 0.07 | 1.20 |
| *Phocaeicola abscessus* | 1.18E-14 | 1.84E-13 | 0.15 | 0.01 | 4.24 |
| *Lachnospiraceae NA* | 3.51E-04 | 1.13E-03 | 0.14 | 0.00 | 5.97 |
| *Selenomonas noxia* | 4.11E-04 | 1.28E-03 | 0.14 | 0.08 | 0.81 |
| *Clostridia UCG-014 NA* | 1.45E-09 | 1.40E-08 | 0.13 | 0.02 | 2.48 |
| *Peptococcus NA* | 1.52E-06 | 8.23E-06 | 0.12 | 0.04 | 1.64 |
| *Lautropia mirabilis* | 1.54E-08 | 1.32E-07 | 0.12 | 0.98 | -3.00 |
| *Prevotella dentalis* | 1.25E-04 | 4.55E-04 | 0.12 | 0.05 | 1.21 |
| *Oribacterium NA* | 7.56E-03 | 1.79E-02 | 0.11 | 0.70 | -2.73 |
| *Family XIII UCG-001 NA* | 9.96E-10 | 1.03E-08 | 0.10 | 0.03 | 1.99 |
| *Butyrivibrio NA* | 5.78E-04 | 1.78E-03 | 0.10 | 0.01 | 3.18 |
| *Capnocytophaga gingivalis* | 1.49E-04 | 5.22E-04 | 0.10 | 0.42 | -2.07 |
| *Alloprevotella rava* | 6.32E-03 | 1.51E-02 | 0.10 | 0.09 | 0.12 |
| *Rothia dentocariosa* | 8.39E-03 | 1.94E-02 | 0.09 | 1.00 | -3.43 |
| *Gemella NA* | 2.76E-03 | 7.31E-03 | 0.09 | 0.32 | -1.81 |
| *Leptotrichia massiliensis* | 1.12E-02 | 2.45E-02 | 0.09 | 0.00 | 10.02 |
| *Prevotella oulorum* | 8.28E-03 | 1.93E-02 | 0.09 | 0.54 | -2.63 |
| *Prevotella melaninogenica* | 4.10E-04 | 1.28E-03 | 0.08 | 0.60 | -2.84 |
| *Megasphaera micronuciformis* | 6.81E-04 | 2.02E-03 | 0.08 | 0.18 | -1.17 |
| *Prevotella veroralis* | 1.79E-06 | 9.47E-06 | 0.07 | 0.84 | -3.52 |
| *Selenomonas artemidis* | 4.40E-03 | 1.14E-02 | 0.07 | 0.28 | -1.98 |
| *Streptococcus gordonii* | 2.09E-03 | 5.65E-03 | 0.07 | 0.05 | 0.58 |
| *Kingella oralis* | 1.18E-06 | 6.85E-06 | 0.07 | 1.02 | -3.85 |
| *Veillonella atypica* | 6.29E-03 | 1.51E-02 | 0.07 | 0.11 | -0.68 |
| *Prevotella maculosa* | 6.81E-07 | 4.24E-06 | 0.07 | 0.34 | -2.34 |
| *Clostridia vadinBB60 group NA* | 9.39E-08 | 7.09E-07 | 0.07 | 0.01 | 3.04 |
| *Rothia NA* | 1.13E-02 | 2.45E-02 | 0.06 | 0.26 | -2.00 |
| *Treponema medium* | 6.30E-03 | 1.51E-02 | 0.06 | 0.00 | 9.55 |
| *Absconditabacteriales (SR1) NA* | 1.20E-08 | 1.07E-07 | 0.06 | 0.00 | 9.44 |
| *Rothia mucilaginosa* | 5.23E-07 | 3.34E-06 | 0.06 | 0.26 | -2.17 |
| *Capnocytophaga sputigena* | 5.93E-05 | 2.42E-04 | 0.05 | 0.10 | -0.93 |
| *Dialister pneumosintes* | 2.73E-05 | 1.19E-04 | 0.05 | 0.06 | -0.08 |
| *Prevotella loescheii* | 6.33E-04 | 1.90E-03 | 0.05 | 0.92 | -4.15 |
| *Veillonella tobetsuensis* | 2.41E-02 | 4.76E-02 | 0.04 | 0.24 | -2.71 |
| *Treponema parvum* | 1.09E-04 | 4.12E-04 | 0.04 | 0.01 | 1.35 |
| *Campylobacter concisus* | 1.47E-06 | 8.16E-06 | 0.04 | 0.19 | -2.40 |
| *Bacteroidales NA* | 1.34E-06 | 7.58E-06 | 0.03 | 0.00 | 4.41 |
| *Actinomyces pacaensis* | 8.22E-03 | 1.93E-02 | 0.03 | 0.00 | 8.52 |
| *Bergeyella NA* | 1.08E-04 | 4.12E-04 | 0.03 | 0.09 | -1.48 |
| *Izemoplasmatales NA* | 2.60E-05 | 1.16E-04 | 0.03 | 0.00 | 8.51 |
| *Erysipelotrichaceae UCG-006 NA* | 1.45E-03 | 4.10E-03 | 0.03 | 0.01 | 2.26 |
| *Gemella sanguinis* | 6.26E-09 | 5.78E-08 | 0.03 | 0.25 | -3.27 |
| *Atopobium parvulum* | 3.55E-04 | 1.13E-03 | 0.02 | 0.14 | -2.69 |
| *Abiotrophia defectiva* | 3.02E-04 | 1.00E-03 | 0.02 | 0.13 | -2.58 |
| *Brachymonas NA* | 2.24E-02 | 4.54E-02 | 0.02 | 0.00 | 7.84 |
| *Pseudoramibacter alactolyticus* | 1.55E-07 | 1.10E-06 | 0.02 | 0.00 | 2.18 |
| *Propionivibrio NA* | 2.36E-02 | 4.73E-02 | 0.02 | 0.00 | 4.14 |
| *Pyramidobacter piscolens* | 2.06E-03 | 5.65E-03 | 0.02 | 0.00 | 7.66 |
| *Prevotella pallens* | 1.01E-04 | 3.99E-04 | 0.02 | 0.05 | -1.65 |
| *Actinobacillus NA* | 1.49E-02 | 3.13E-02 | 0.01 | 0.04 | -1.38 |
| *Leptotrichia goodfellowii* | 7.80E-04 | 2.26E-03 | 0.01 | 0.41 | -4.87 |
| *Prevotella salivae* | 5.04E-07 | 3.34E-06 | 0.01 | 0.20 | -4.07 |
| *Campylobacter showae* | 7.44E-07 | 4.41E-06 | 0.01 | 0.05 | -1.99 |
| *Veillonella dispar* | 1.00E-05 | 4.89E-05 | 0.01 | 0.16 | -3.80 |
| *Capnocytophaga haemolytica* | 2.43E-04 | 8.30E-04 | 0.01 | 0.12 | -3.42 |
| *Streptococcus sanguinis* | 4.67E-03 | 1.20E-02 | 0.01 | 0.16 | -4.30 |
| *Veillonella rogosae* | 1.73E-02 | 3.55E-02 | 0.01 | 0.08 | -3.37 |
| *Actinomyces gerencseriae* | 7.33E-07 | 4.41E-06 | 0.01 | 0.14 | -4.27 |
| *Pseudopropionibacterium propionicum* | 1.33E-04 | 4.74E-04 | 0.01 | 0.03 | -2.29 |
| *Prevotellaceae NA* | 1.37E-02 | 2.94E-02 | 0.01 | 0.01 | -1.02 |
| *Streptococcus massiliensis* | 4.29E-05 | 1.81E-04 | 0.00 | 0.02 | -1.79 |
| *Oribacterium sinus* | 1.17E-03 | 3.36E-03 | 0.00 | 0.04 | -3.59 |
| *Streptococcus mutans* | 1.64E-03 | 4.59E-03 | 0.00 | 0.09 | -5.18 |
| *Haemophilus parainfluenzae* | 1.51E-04 | 5.23E-04 | 0.00 | 0.30 | -6.99 |
| *Filifactor NA* | 9.22E-03 | 2.09E-02 | 0.00 | 0.00 | -1.49 |
| *Peptoniphilus lacrimalis* | 5.21E-05 | 2.16E-04 | 0.00 | 0.00 | -1.28 |
| *Bacteroidia NA* | 1.00E-02 | 2.23E-02 | 0.00 | 0.00 | 1.00 |
| *Lautropia NA* | 2.58E-05 | 1.16E-04 | 0.00 | 0.00 | -1.89 |
| *Conservatibacter NA* | 2.36E-03 | 6.33E-03 | 0.00 | 0.00 | -0.88 |
| *Prevotella nanceiensis* | 1.52E-07 | 1.10E-06 | 0.00 | 0.04 | -7.36 |
| *Staphylococcus NA* | 5.23E-03 | 1.33E-02 | 0.00 | 1.63 | -14.22 |
| *Prevotella histicola* | 7.33E-04 | 2.15E-03 | 0.00 | 0.11 | -10.27 |
| *Actinobacillus pleuropneumoniae* | 1.12E-04 | 4.18E-04 | 0.00 | 0.04 | -8.88 |
| *Haemophilus sputorum* | 3.09E-04 | 1.01E-03 | 0.00 | 0.04 | -8.71 |
| *Streptococcus parasanguinis* | 1.28E-05 | 6.00E-05 | 0.00 | 0.01 | -7.07 |
| *Gemella parahaemolysans* | 3.04E-05 | 1.31E-04 | 0.00 | 0.01 | -6.38 |
| *Micrococcales NA* | 1.66E-05 | 7.64E-05 | 0.00 | 0.00 | -3.80 |
