# Supplementary material for: Personalized antibiotic selection in periodontal treatment improves clinical and microbiological outputs
Source: Front Cell Infect Microbiol. 2023 Dec 18;13:1307380. doi: 10.3389/fcimb.2023.1307380 (PMC10765594; doi:10.3389/fcimb.2023.1307380)
Supplement: Supplementary Figure 1 — Workflow of experimental set up to evaluate transport media selection and growing conditions. Subgingival plaque samples (n=5) were collected and placed into RTF, VMGIII and VMG III Agar transport media using 10 sterile paper points and stored at room temperature for 0, 24 and 48h. After that, periodontal biofilms were grown in the impedance xCELLigence system, which measures biofilm growth in real-time. Formed biofilms were collected at 8h and 20h for DNA extraction and 16S rRNA gene Illumina sequencing to compare biofilm bacterial composition to the initial subgingival sample inoculum. [file DataSheet_1.pdf]

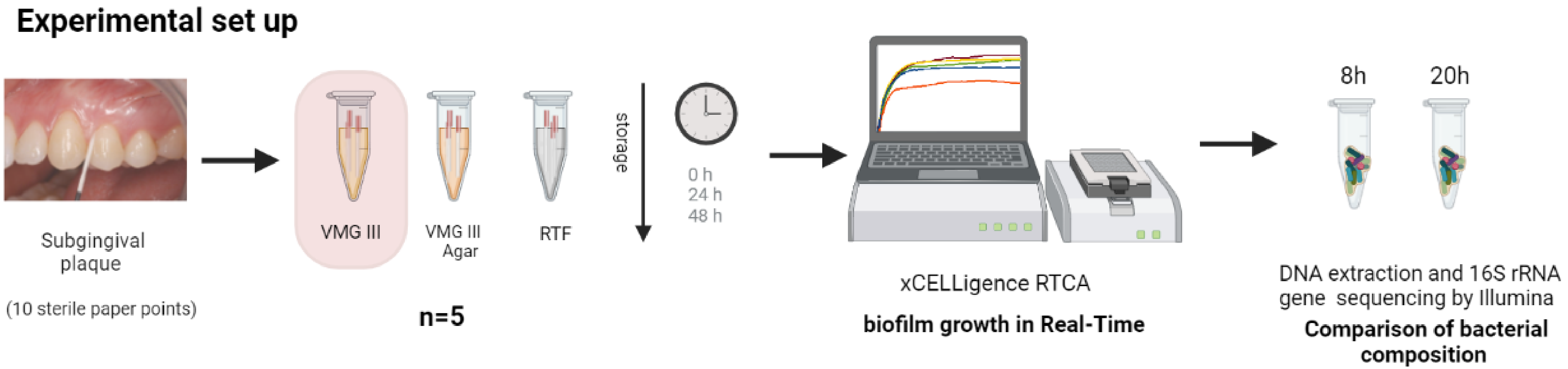
Experimental set up
Subgingival plaque
(10 sterile paper points)
VMG III
VMG III Agar
RTF
n=5
storage
0 h
24 h
48 h
xCELLigence RTCA
biofilm growth in Real-Time
8h
20h
DNA extraction and 16S rRNA gene sequencing by Illumina
Comparison of bacterial composition

Figure S1.

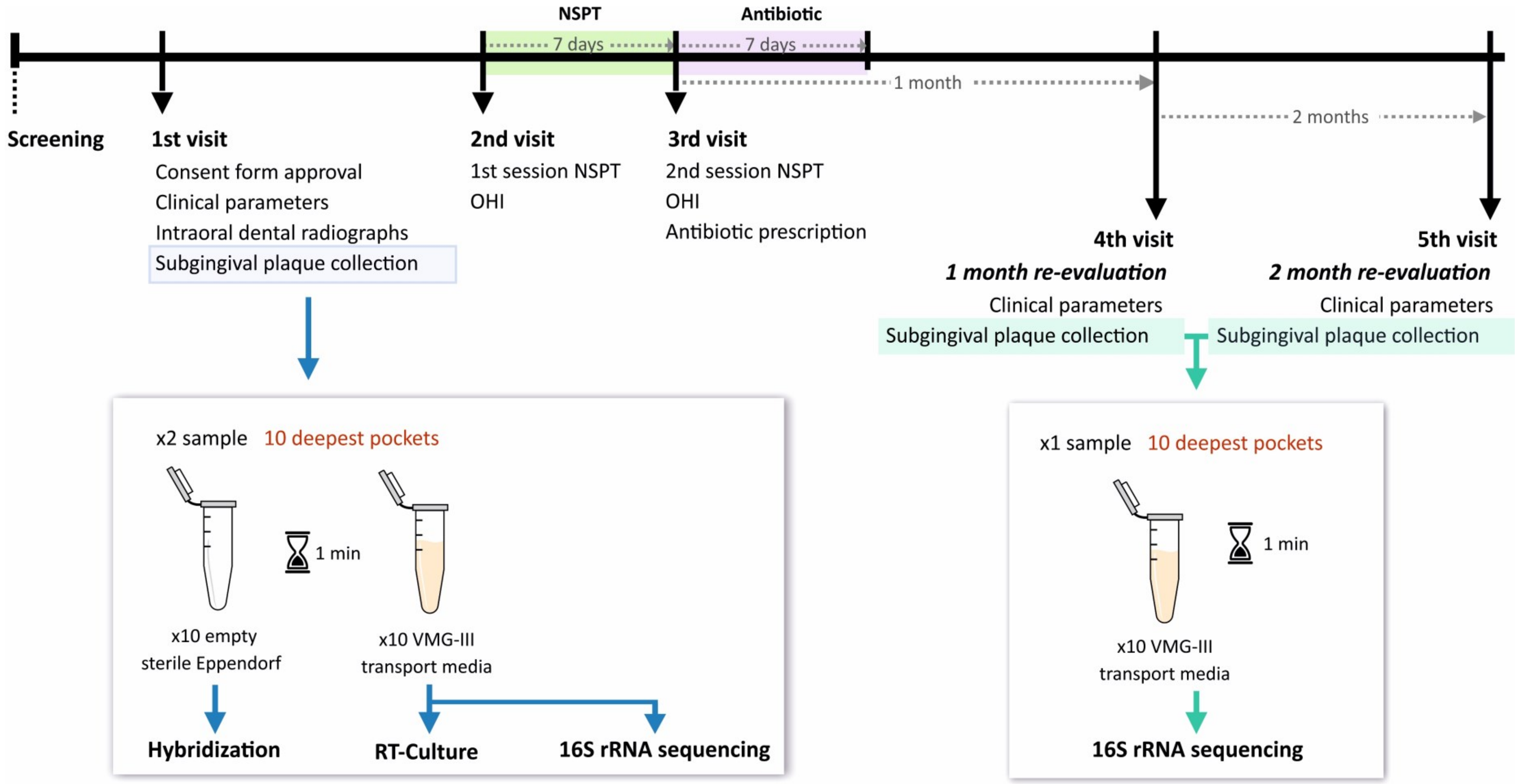
NSPT
Antibiotic
7 days
7 days
1 month
2 months
Screening
1st visit
Consent form approval
Clinical parameters
Intraoral dental radiographs
Subgingival plaque collection
2nd visit
1st session NSPT
OHI
3rd visit
2nd session NSPT
OHI
Antibiotic prescription
4th visit
1 month re-evaluation
Clinical parameters
Subgingival plaque collection
5th visit
2 month re-evaluation
Clinical parameters
Subgingival plaque collection
x2 sample 10 deepest pockets
1 min
x10 empty
sterile Eppendorf
x10 VMG-III
transport media
Hybridization
RT-Culture
16S rRNA sequencing
x1 sample 10 deepest pockets
1 min
x10 VMG-III
transport media
16S rRNA sequencing

Figure S2.

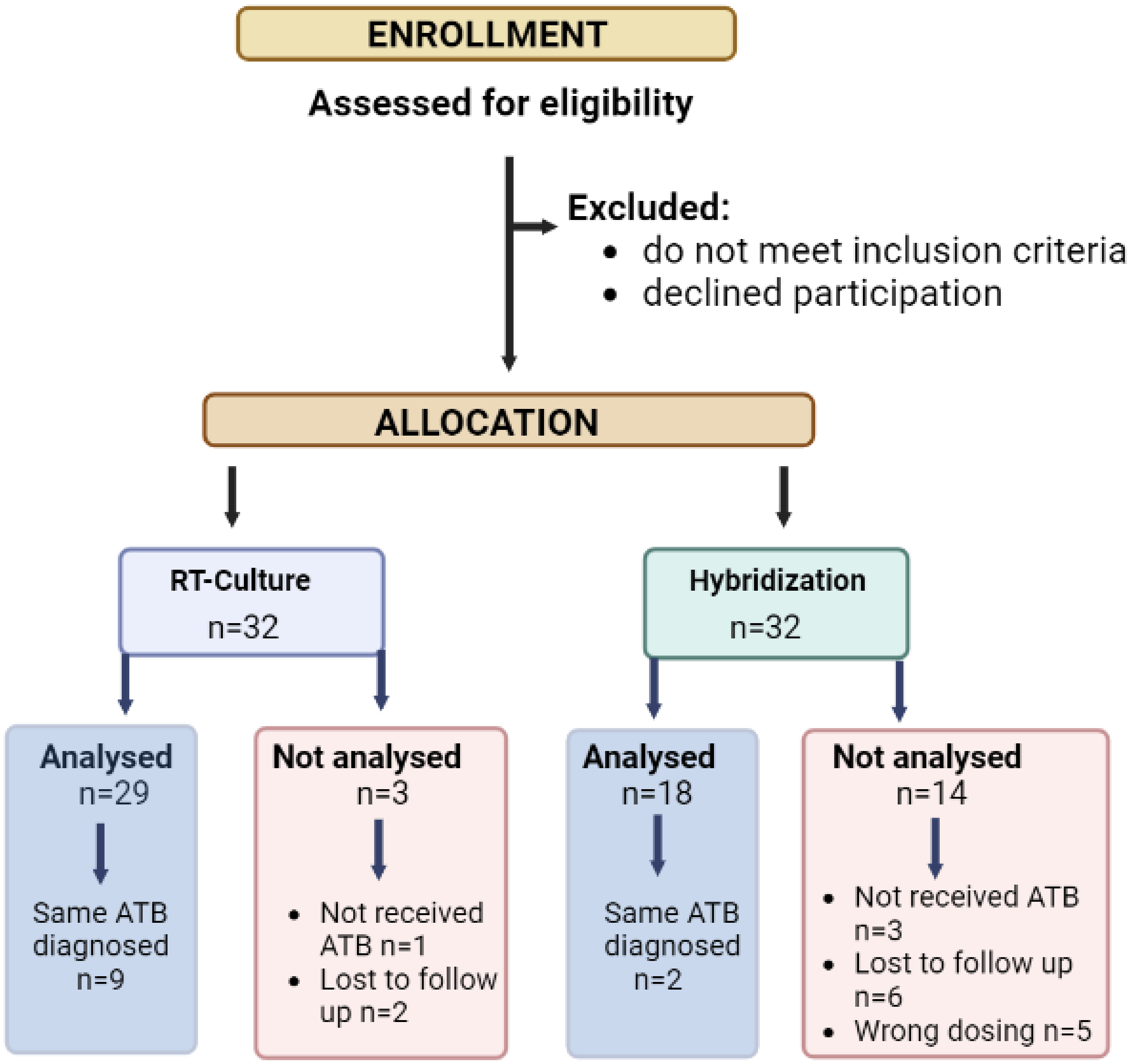
ENROLLMENT
Assessed for eligibility
Excluded:
• do not meet inclusion criteria
• declined participation
ALLOCATION
RT-Culture
n=32
Hybridization
n=32
Analysed
n=29
Same ATB diagnosed n=9
Not analysed
n=3
• Not received ATB n=1
• Lost to follow up n=2
Analysed
n=18
Same ATB diagnosed n=2
Not analysed
n=14
• Not received ATB n=3
• Lost to follow up n=6
• Wrong dosing n=5

Figure S3.

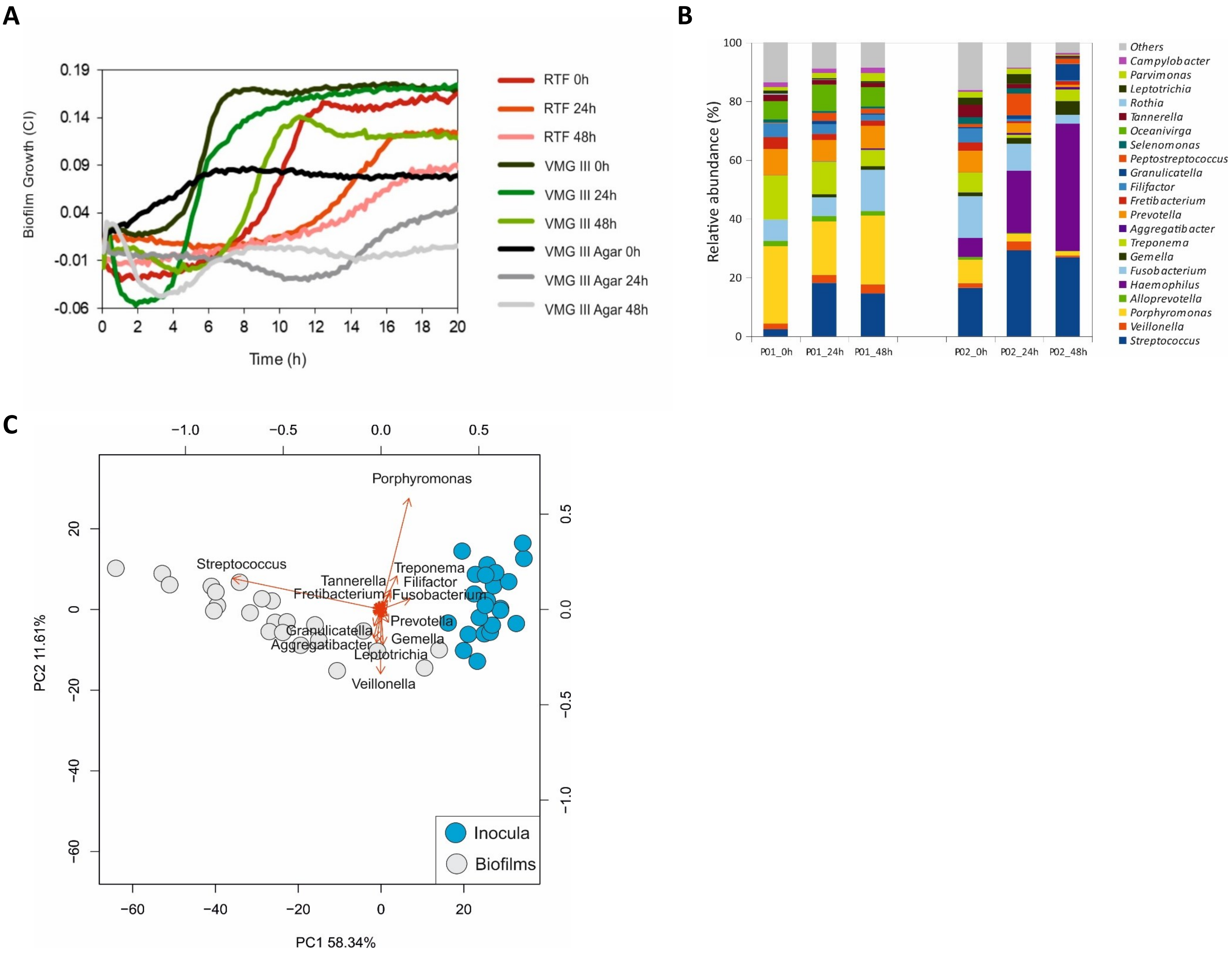
A
B
C
Biofilm Growth (CI)
Time (h)
RTF 0h
RTF 24h
RTF 48h
VMG III 0h
VMG III 24h
VMG III 48h
VMG III Agar 0h
VMG III Agar 24h
VMG III Agar 48h
Relative abundance (%)
P01_0h
P01_24h
P01_48h
P02_0h
P02_24h
P02_48h
Others
Campylobacter
Parvimonas
Leptotrichia
Rothia
Tannerella
Oceanivirga
Selenomonas
Peptostreptococcus
Granulicatella
Filifactor
Fretibacterium
Prevotella
Aggregatibacter
Treponema
Gemella
Fusobacterium
Haemophilus
Alloprevotella
Porphyromonas
Veillonella
Streptococcus
Porphyromonas
Streptococcus
Tannerella
Treponema
Filifactor
Fretibacterium
Fusobacterium
Prevotella
Granulicatella
Aggregatibacter
Gemella
Leptotrichia
Veillonella
Inocula
Biofilms
PC2 11.61%
PC1 58.34%

Figure S4.

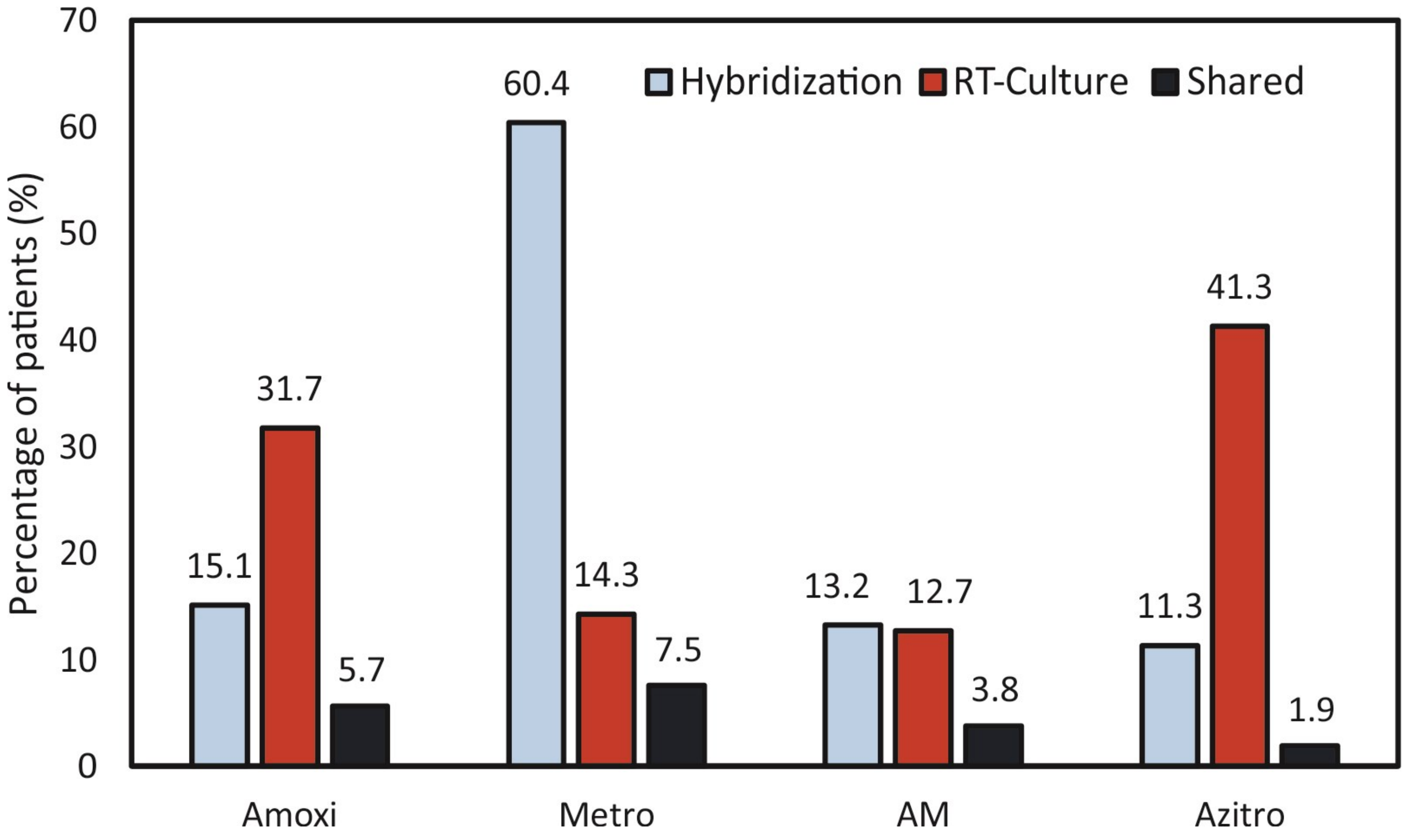
Hybridization
RT-Culture
Shared
Percentage of patients (%)
70
60
50
40
30
20
10
0
60.4
31.7
41.3
15.1
5.7
14.3
7.5
13.2
12.7
3.8
11.3
1.9
Amoxi
Metro
AM
Azitro

Figure S5.

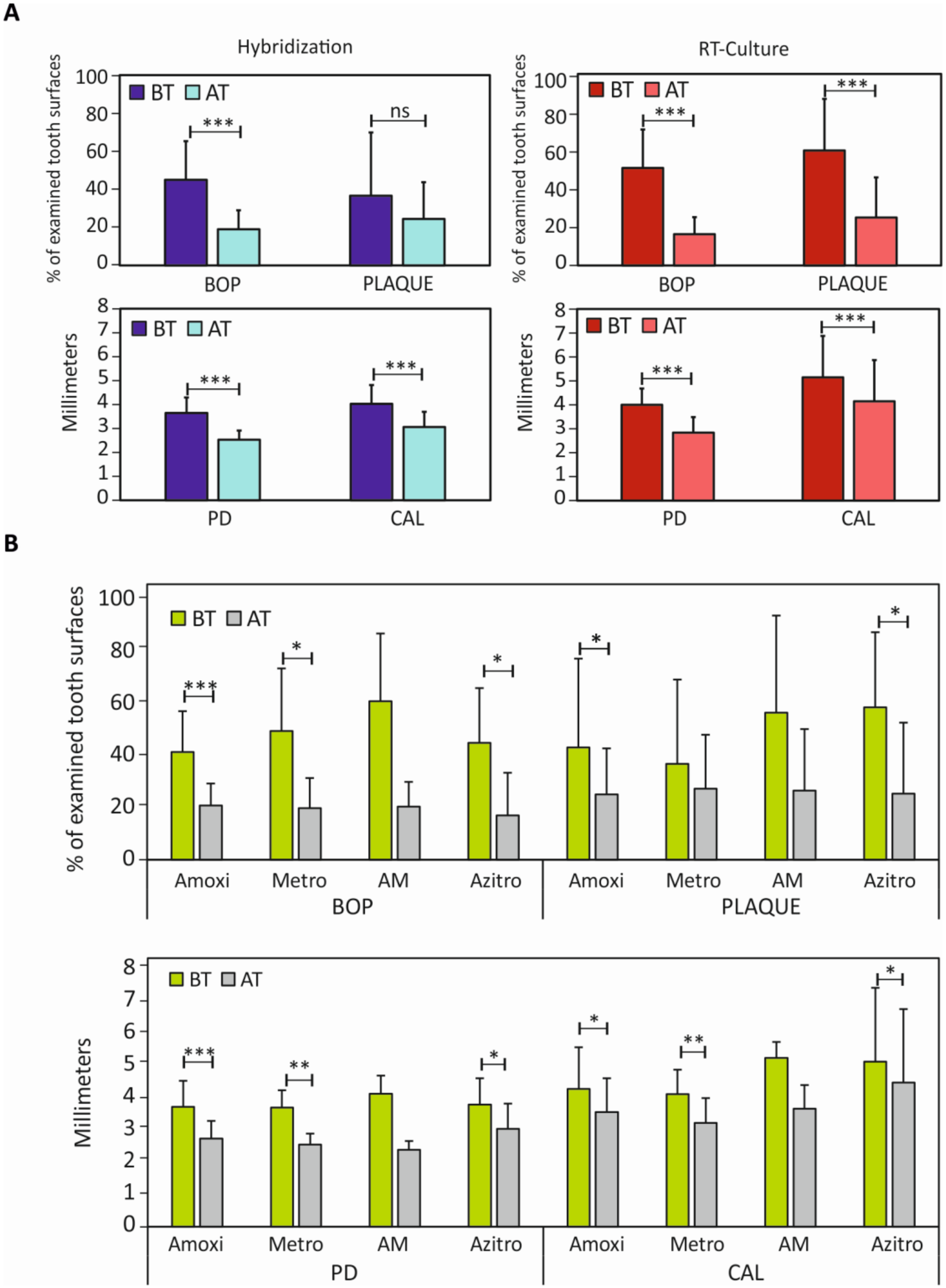
A
Hybridization
RT-Culture
% of examined tooth surfaces
BT
AT
***
ns
BOP
PLAQUE
Millimeters
PD
CAL
B
Amoxi
Metro
AM
Azitro
**
*

Figure S6.

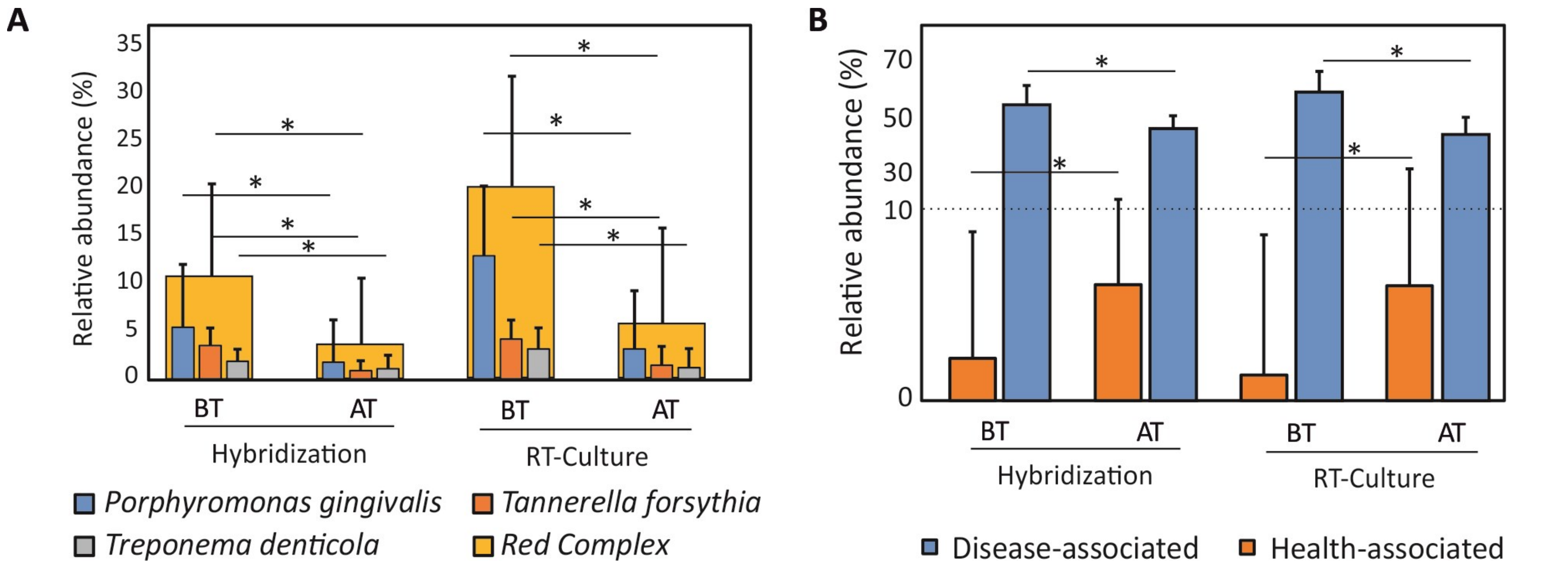
A
Relative abundance (%)
35
30
25
20
15
10
5
0
*
BT
AT
Hybridization
BT
AT
RT-Culture
Porphyromonas gingivalis
Tannerella forsythia
Treponema denticola
Red Complex
B
Relative abundance (%)
70
50
30
10
0
BT
AT
Hybridization
BT
AT
RT-Culture
Disease-associated
Health-associated

Figure S7.

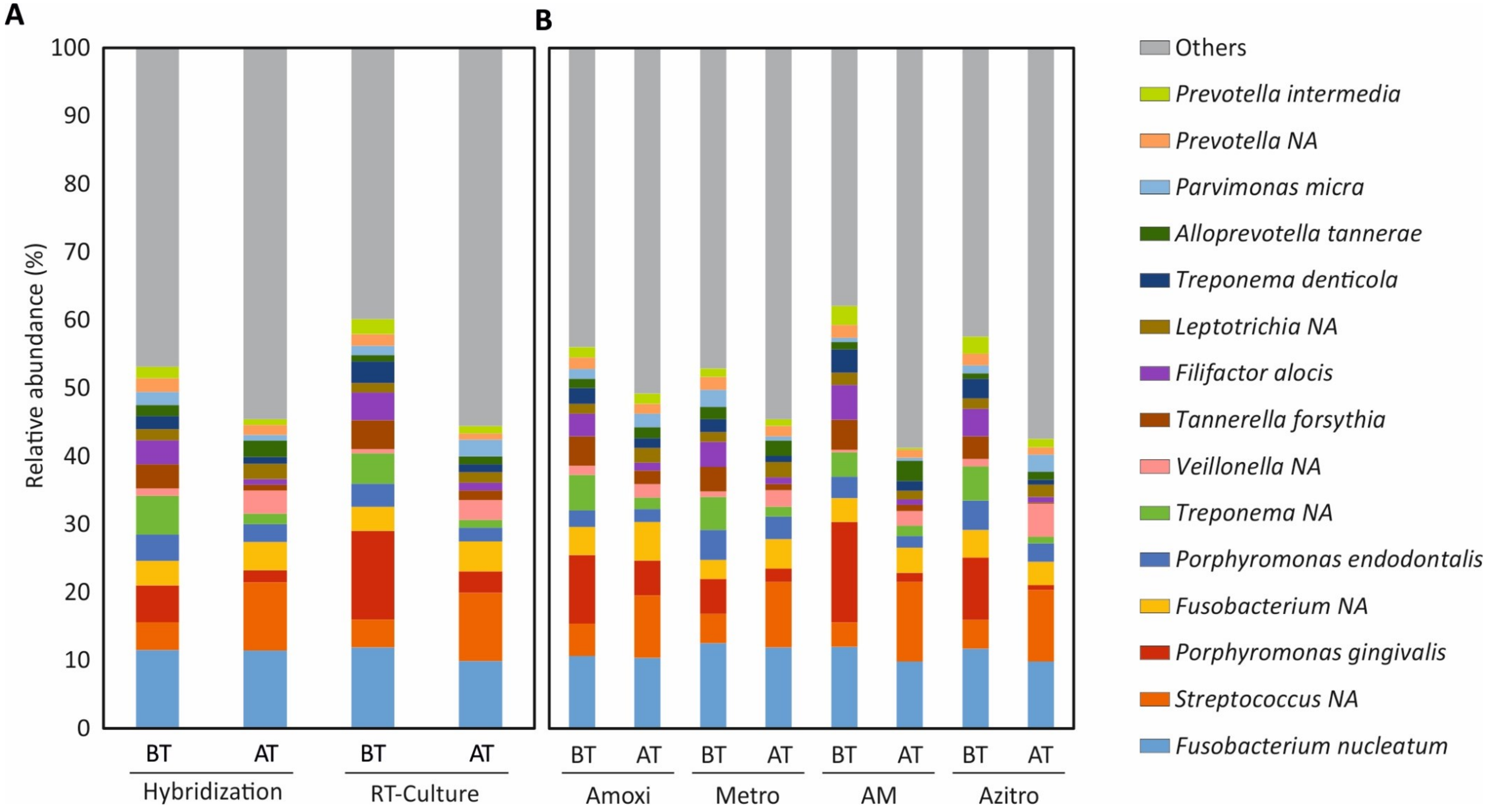
A
B
Relative abundance (%)
100
90
80
70
60
50
40
30
20
10
0
BT
AT
BT
AT
Hybridization
RT-Culture
BT
AT
BT
AT
BT
AT
BT
AT
Amoxi
Metro
AM
Azitro
Others
Prevotella intermedia
Prevotella NA
Parvimonas micra
Alloprevotella tannerae
Treponema denticola
Leptotrichia NA
Filifactor alocis
Tannerella forsythia
Veillonella NA
Treponema NA
Porphyromonas endodontalis
Fusobacterium NA
Porphyromonas gingivalis
Streptococcus NA
Fusobacterium nucleatum

Figure S8.

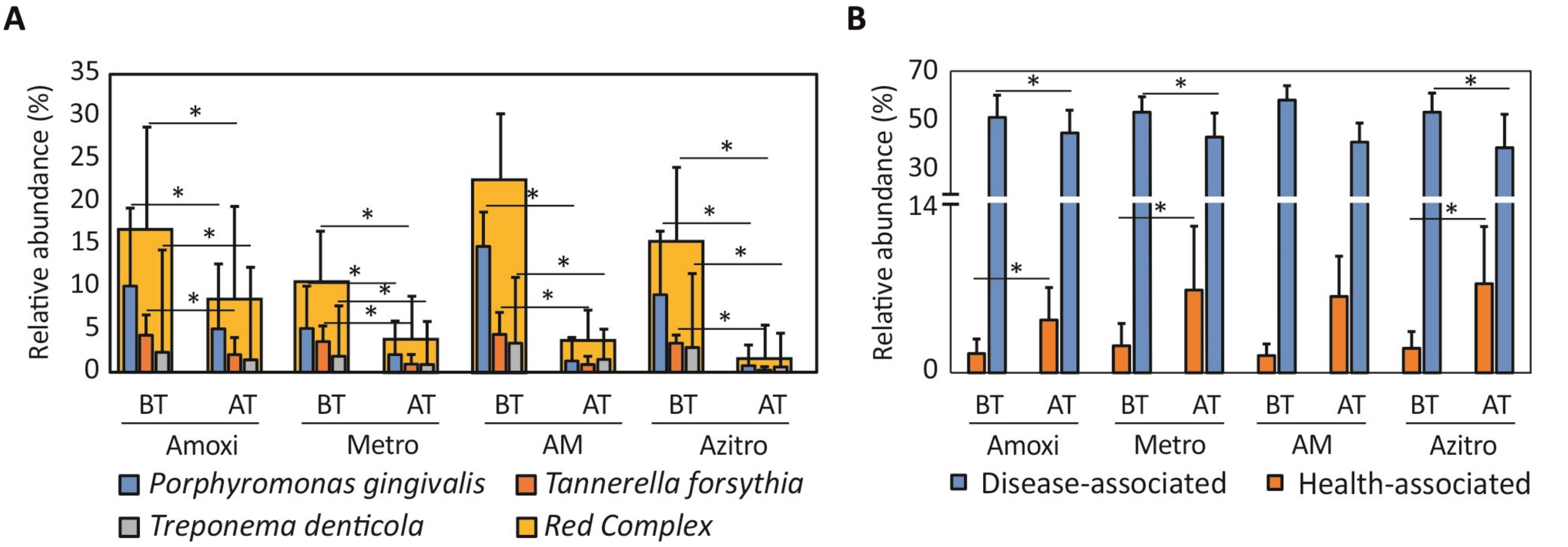
A
B
Relative abundance (%)
35
30
25
20
15
10
5
0
BT
AT
Amoxi
Metro
AM
Azitro
Porphyromonas gingivalis
Tannerella forsythia
Treponema denticola
Red Complex
70
50
30
14
Disease-associated
Health-associated

Figure S9.

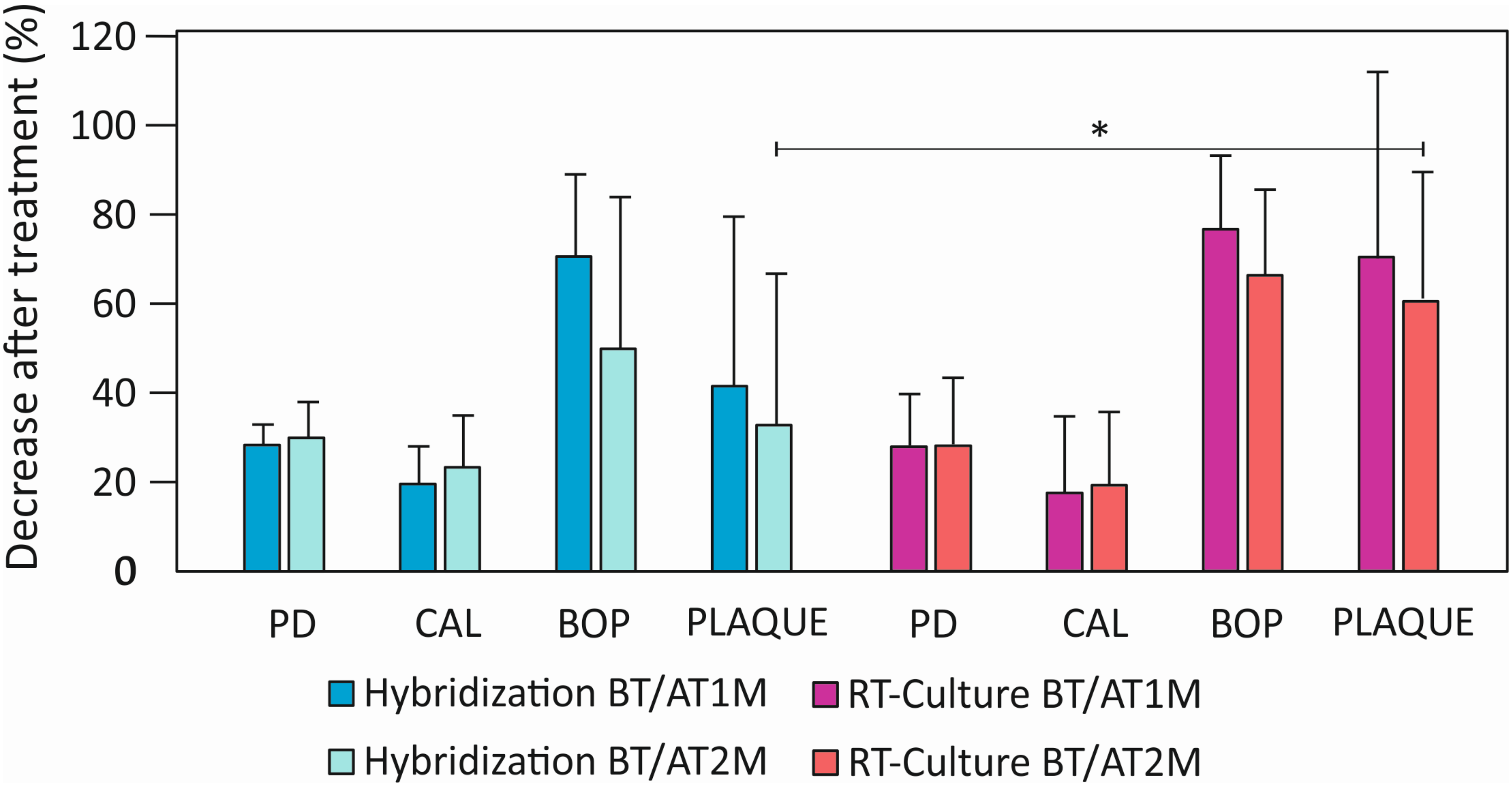
Decrease after treatment (%)
120
100
80
60
40
20
0
*
PD
CAL
BOP
PLAQUE
PD
CAL
BOP
PLAQUE
Hybridization BT/AT1M
RT-Culture BT/AT1M
Hybridization BT/AT2M
RT-Culture BT/AT2M

Figure S10.

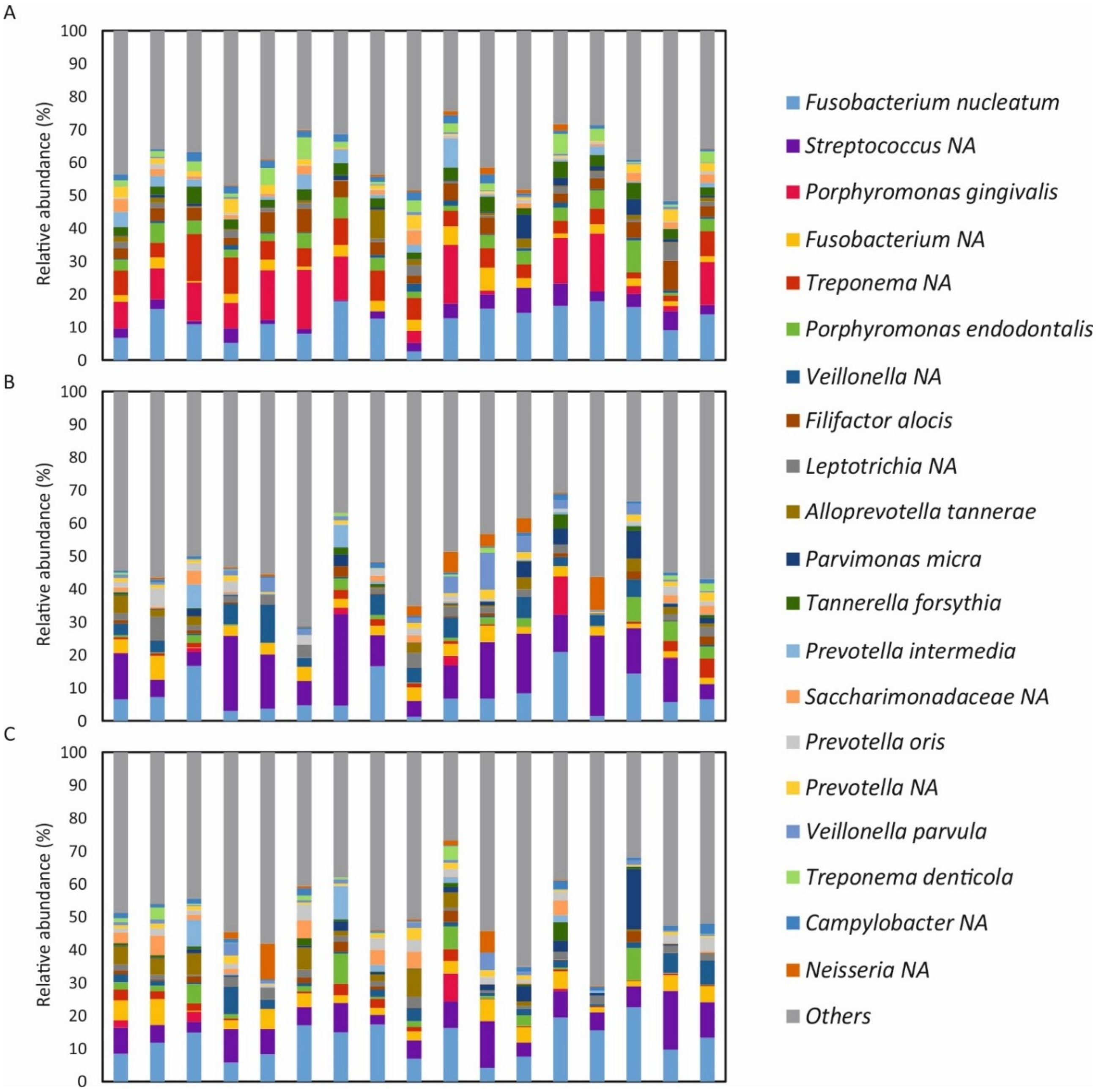
A
B
C
Relative abundance (%)
0
10
20
30
40
50
60
70
80
90
100
Fusobacterium nucleatum
Streptococcus NA
Porphyromonas gingivalis
Fusobacterium NA
Treponema NA
Porphyromonas endodontalis
Veillonella NA
Filifactor alocis
Leptotrichia NA
Alloprevotella tannerae
Parvimonas micra
Tannerella forsythia
Prevotella intermedia
Saccharimonadaceae NA
Prevotella oris
Prevotella NA
Veillonella parvula
Treponema denticola
Campylobacter NA
Neisseria NA
Others

Figure S11.

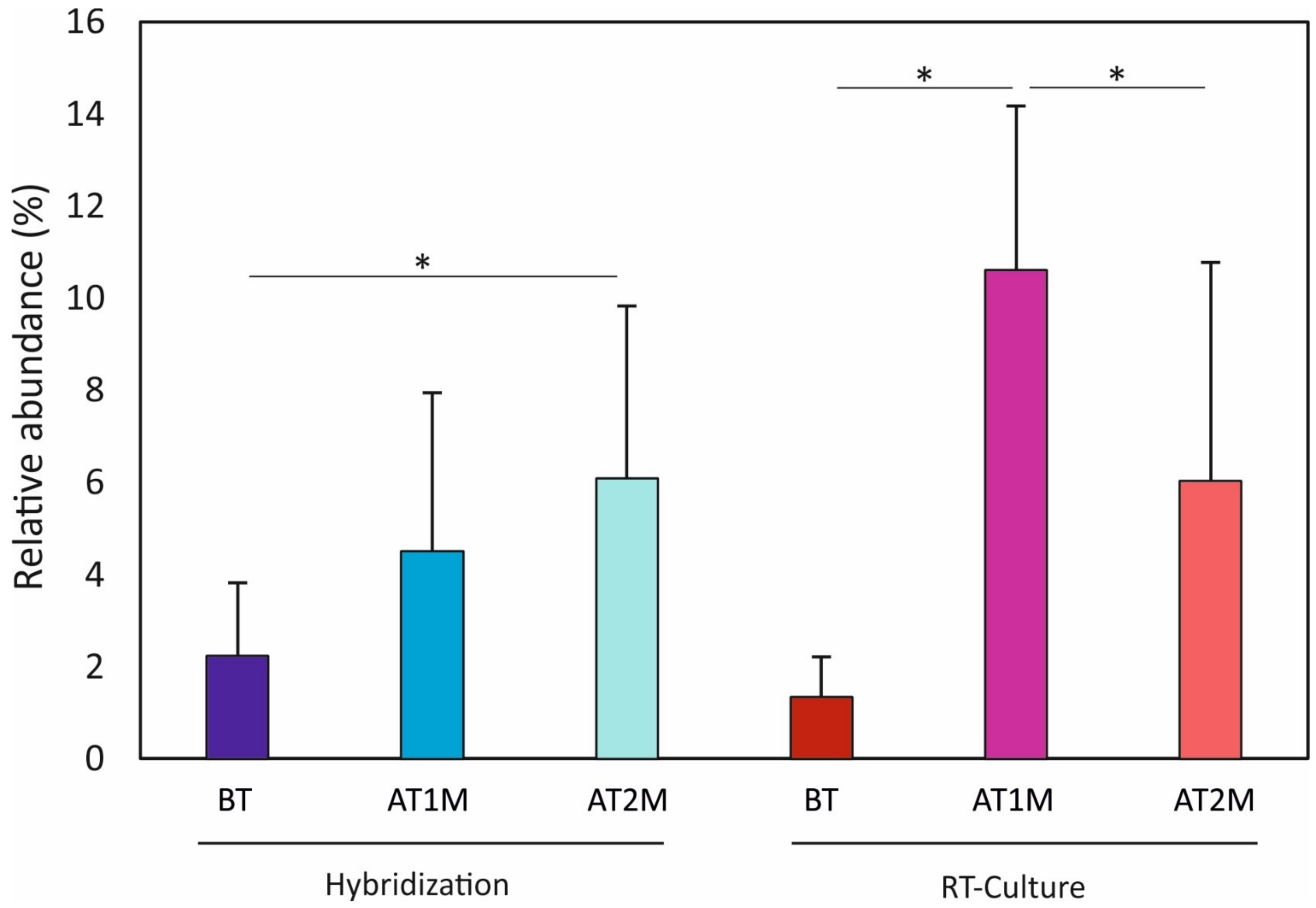
16
14
12
10
8
6
4
2
0
Relative abundance (%)
*
*
*
BT
AT1M
AT2M
BT
AT1M
AT2M
Hybridization
RT-Culture

Figure S12.
